# Novel *Aeromonas* Phage Ahy-Yong1 and Its Protective Effects against *Aeromonas hydrophila* in Brocade Carp (*Cyprinus aka* Koi)

**DOI:** 10.3390/v14112498

**Published:** 2022-11-11

**Authors:** Lingting Pan, Dengfeng Li, Wei Lin, Wencai Liu, Chenxin Qu, Minhua Qian, Ruqian Cai, Qin Zhou, Fei Wang, Yigang Tong

**Affiliations:** 1 Key Laboratory of Marine Biotechnology of Zhejiang Province, School of Marine Sciences, Ningbo University, Ningbo 315211, China; 2 College of Life Science and Technology, Beijing University of Chemical Technology, Beijing 100029, China

**Keywords:** *Aeromonas hydrophila*, phage, therapy, brocade carp, genome

## Abstract

*Aeromonas hydrophila* is a zoonotic pathogen and an important fish pathogen. A new lytic phage, Ahy-yong1, against multi-antibiotic-resistant pathogen *A. hydrophila* was isolated, identified, and tentatively used in therapy. Ahy-yong1 possesses a head of approximately 66 nm in diameter and a short tail of approximately 26 nm in length and 32 nm in width. Its complete dsDNA genome is 43,374 bp with a G + C content of 59.4%, containing 52 predicted opening reading frames (ORFs). Taxonomic analysis indicated Ahy-yong1 as a new species of the *Ahphunavirus* genus of the *Autographiviridae* family of the *Caudoviricetes* class. Ahy-yong1 was active only against its indicator host strain among the 35 strains tested. It is stable at 30–40 °C and at pH 2–12. *Aeromonas* phage Ahy-yong1 revealed an effective biofilm removal capacity and an obvious protective effect in brocade carp (*Cyprinus aka* Koi). The average cumulative mortality for the brocade carp in the blank groups intraperitoneally injected with PBS was 1.7% ± 2.4%;for the control groups treated with *A. hydrophila* (10^8^ CFU/fish) via intraperitoneal injection, it was 100.00%;and for the test group I, successively treated with *A. hydrophila* (10^8^ CFU/fish) and *Aeromonas* phage Ahy-yong1 (10^7^ PFU/fish) via intraperitoneal injection witha time interval of 2 hours, it was only 43.4% ± 4.7%. Furthermore, the cumulative mortality of the test group II, successively treated with *Aeromonas* phage Ahy-yong1 (10^7^ PFU/fish) and *A. hydrophila* (10^8^ CFU/fish), was only 20.0% ± 8.2%, and that of the test group III, simultaneously treated with *Aeromonas* phage Ahy-yong1 (10^7^ PFU/fish) and *A. hydrophila* (10^8^ CFU/fish), was only 30.0% ± 8.2%. The results demonstrated that phage Ahy-yong1 was very effective in the therapies against *A. hydrophila* A18, prophylaxis was more effective than rescue, and earlier treatment was better for the reduction of mortality. This study enriches knowledge about *Aeromonas* phages.

## 1. Introduction

In the 21st century, bacterial infections are still global threats to human and animal life. Antibiotic-resistant bacteria are becoming increasingly widespread with the abuse of antibiotics, which runs the risk of forcing us into the “post antibiotic era”. Hundreds of tons of antimicrobials are used annually in aquaculture [1]. The rising drug resistance in aquatic bacteria promotes aquatic environments to act as a gateway for antimicrobial resistance [2]. These cause a consensus that the application of antibiotics to prevent fish diseases should not be a primary treatment option in fish farming practices [3]. The search for alternative environmentally friendly approaches is urgently needed [1]. Phage therapy, a technique of using bacteriophages to treat bacterial infections, has attracted wide attention in recent years. Phage therapy is currently considered a best approach for the control of bacterial infections in aquaculture [3]. In the past decades, a number of reports have proven the feasibility of phage therapy in the treatment of bacterial infections for human and animals [4,5,6,7]. To date, phage therapy studies and practices mainly focused on lytic phages of the *Caudoviricetes* class [8].

Lytic phage isolation and therapy study is necessary to control *Aeromonas* diseases. The genus *Aeromonas* belongs to the family *Aeromonadaceae*, order *Aeromonadales*, class *Gammaproteobacterias* [9]. They are Gram-negative bacilli normally found in aquatic environments and food products. Some species of genus *Aeromonas* are pathogenic to human and aquatic animals [9,10]. There are numerous reports on diseases associated with *Aeromonas*. Bacteriosis of freshwater fish in the natural environment and in fish farms is mainly caused by *Aeromonas* species [3,11]. *Aeromonas hydrophila* is a zoonotic pathogen and an important fish pathogen frequently causing ulcers, hemorrhages, furunculosis, and septicemia [12,13,14]. *A. hydrophila* diseases do serious harm to the development of aquaculture industry. *A. hydrophila* is capable of forming biofilms and is found to carry antibiotic-resistance genes [15,16]. These two features are both advantageous for it to persist in antibiotic treatment. The biological control strategy by isolating and using phages is worth advocating.

Although there are 69 *A. hydrophila* phage genomes in the public databases, there are few studies on the biological characteristics of them. So far, 10 *A.hydrophila* phages have been studied for the treatment of fish diseases caused by *A.hydrophila*. These phages include PZL-Ah1 [17], pAh1 [18], pAh1-C [19], pAh6-C [19], pAh6.2TG [20], Akh-2 [21], AhMtk13a [3], PVN02 [22], Φ2 [23], and Φ5 [23]. They all improved fish survival in *A. hydrophila* infection. For example, in the therapy test of pAh-1 (at MOI of 10) against *A. hydrophila* (2 × 10^6^ CFU per fish), the cumulative zebrafish mortality rates of the therapy groups and non-treatment groups were, respectively, 56.7 ± 35.1% and 96.7 ± 5.8%. In China, *A. hydrophila* phages were isolated from water, fish guts, the intestinal tracts of Chinese soft-shell turtle (*Pelodiscussinensis*), and so on [24,25,26,27,28,29]. Zhang’s study found that *A. hydrophila* PZL-Ah1 could effectively protect crucian carp against *A. hydrophila* [17]. In the experiment of Huo et al., phage treatment of crayfish (*Procambarusclarkii*) against *A. hydrophila* showed protection rates of 66% and 20%, respectively, by injection and immersion [26].

Most *Aeromonas* phages showed narrow host specificity [30]. A narrow host range can be disadvantageous when designing therapeutic or biocontrol applications utilizing a single phage. Phage cocktails may be optimal. Too few phages were studied concerning phage therapy in fish against *A. hydrophila*. To lay the material and theoretical foundation for antibiotic replacement therapy, additional phages need to be isolated, and more research focused upon their application in aquaculture needs to be conducted.

In this study, a new lytic phage against multiantibiotic-resistant *A. hydrophila* was isolated, identified, and the effects of application were assessed in an infection model utilizing brocade carp.

## 2. Materials and Methods

### 2.1. Bacterial Strain

The bacterial strain *A. hydrophila* A18, kindly provided by Phagelux Biotechnology Co., Ltd (Nanjing, China), was isolated from Nanjing Zhongcai wuliu Aquatic Product Market of Jiangsu province. It was grown in Tryptic Soy Broth (TSB) medium (tryptone 17 g/L, phytone 3 g/L, NaCl 5 g/L, K_2_HPO_4_ 2.5 g/L, glucose 2.5 g/L, pH 7.2) at 29 °C with shaking at 180 rpm.

### 2.2. Antibiotic Susceptibility Test

The antibiotic susceptibility test for *A. hydrophila* A18 was conducted using antibiological susceptibility disks (Hangzhou Binhe Microorganism Reagent. Co., Ltd., Hangzhou, China), following the instructions of the manufacturer disks and in the light of the standard of the Clinical and Laboratory Standards Institute (CLSI). Sixteen kinds of antibiotic (including penicillin G, amoxicillin, cephalexin, kanamycin, gentamicin, tobramycin, azithromycin, chloramphenicol, ofloxacin, tetracycline, doxycycline, rifampicin, compound sulfamethoxazole, vancomycin, polymyxin B, and clindamycin) disks were used. *A. hydrophila* A18 culture in the exponential growth phase (1.5 × 10^8^ CFU mL^−1^) were spread by sterile swab on Muller–Hinton agar plates. After drying at room temperature for 5 min, antibiotic discs were placed on the surface of the medium. The plates were then incubated at 29 °C for 16 to 18 h. The diameters of the formed inhibition zones were measured.

### 2.3. Phage Isolation and Purification

A surface water sample used for phage isolation was collected (on 5 March 2021) from a stream (North latitude, 29.764532; East longitude, 121.907201) in Chunxiao Town of Ningbo City in Zhejiang province, China. After centrifugation at 10,000 g for 10 min at 4 °C, 40 mL of the supernatant, 20 mL of 3 × TSB medium, and 1 mL of *A. hydrophila* A18 culture in the exponential growth phase (OD_600_ ≈ 0.6, 1.82 × 10^8^ CFU/mL) were mixed. The mixtures were incubated at 29 °C with a shaking speed of 180 rpm for 3 h to proliferate phages. The culture was centrifuged at 10,000× *g* for 10 min at 4 °C, and the supernatant was filtered sequentially through 0.45 μm and 0.22 μm microporous filters to remove particles and bacterial cells. The filtrate was taken to purify the phage strain via three successive single-plaque isolations using the conventional double-layer agar method [31].

### 2.4. Phage Morphology

For electron microscopy observation, 1 mL of phage lysate was centrifuged at 10,000× *g* for 10 min at 4 °C, the supernatant was laid on sucrose density gradient (20–40%) and centrifuged at 58,000× *g* for 1 h. The sediment was washed twice with 0.01 M phosphate buffered saline (PBS) and resuspended in 200 μL of PBS. The phage suspension and *A. hydrophila* A18 culture infected with *Aeromonas* phage Ahy-yong1 (abbreviated as Ahy-yong1) were deposited, respectively, on carbon-coated copper grids for 10 min, stained with 3% uranyl acetate for 20 s, and observed under a transmission electron microscope (TEM) (Hitachi-7650, Tokyo, Japan).

### 2.5. Host Range Test

Thirty-five bacterial strains (Table 1) were used to test the host range of *Aeromonas* phage Ahy-yong1 using the spot test as described [32]. Each 200 µL of tested bacterial cells was added to 4 mL of melted 0.7% agar medium (pre-incubated at 42 °C), mixed quickly and poured onto 1.5% solid agar medium to make double-layered agar plates. Each 5 µL of phage suspension (3.2 × 10^10^ PFU/mL) was spotted onto the solidified double-layer plate, respectively, in triplicate. The plates were incubated at 29 °C overnight. Clear zones at the spot areas indicated positive susceptibility. The above tests were repeated three times. *Aeromonas* phage Ahy-yong1 could only lyse the indicator bacterium. In order to test the infective activity of Ahy-yong1 against the tested bacteria at 37 °C, a similar experiment was carried out with the difference that the plates were incubated at 37 °C. In addition, tenfold series gradient dilutions (10^0^–10^−3^) of Ahy-yong1 suspension (3.2 × 10^10^ PFU/mL) were spotted onto the double-layer plate, respectively, and incubated at 37 °C overnight.

### 2.6. Optimal Multiplicity of Infection (MOI), Optimum Adsorption Time, and One-Step Growth Curve

The multiplicity of infection (MOI) is the ratio of the number of phage particles to the host bacteria cells [33]. To clarify the optimal MOI of *Aeromonas* phage Ahy-yong1, 3.18 × 10^7^ CFU of the host bacteria *A. hydrophila* A18 in the logarithmic phase were mixed with a set of serial dilutions of *Aeromonas* phage Ahy-yong1 suspension at MOIs of 0.0001, 0.001, 0.01, and 0.1, respectively, in triplicate. After absorption for 10 min at 29 °C, the mixtures were centrifuged at 10,000× *g* for 10 min to remove supernatant. The sediments were washed twice with TSB, resuspended in 5 mL TSB medium, and incubated at 29 °C for 4 h with a shaking speed of 180 rpm. The titers of the collected samples were measured by using the double-layer agar plate method [31]. The experiment was repeated three times. The MOI resulting in the highest phage titer was considered the optimal MOI.

To clarify the optimum adsorption time of *Aeromonas* phage Ahy-yong1, the phage was mixed with a logarithmic-phaseA18 culture at the optimal MOI of 0.1 in triplicate and incubated at 29 °C with a shaking speed of 180 rpm. Samples were collected at 0, 2, 4, 6, 8, 10, 15, and 20 min post-inoculation and centrifuged at 10,000× *g* for 10 min at 4 °C. Phage titers of the supernatants were measured by using the double-layer agar plate method. The adsorption rate was calculated by the ratio of the difference between average phage titers at 0 min and the average phage titers at each sampling time to the average phage titer at 0 min. The optimal adsorption time of phage is the shortest time need to reached high adsorption rate.

Next, 400 μL of phage Ahy-yong1 suspension (3.18 × 10^8^ PFU/mL) was mixed with 40 mL of logarithmic-phase *A. hydrophila* A18 culture (3.18 × 10^7^ CFU/mL) at the proposed optimal MOI of 0.1 in triplicate and allowed to absorb at 29 °C for 2 min (the optimum adsorption time). The non-adsorbed phages were removed by centrifugation at 10,000× *g* for 10 min. The sediment was washed twice with fresh TSB medium, suspended in an equal volume of TSB medium, and then incubated at 29 °C with a shaking speed of 180 rpm. The phage titer at 10, 20, 30, 40, 50, 60, 90, 120, 150, 180, 210, and 240 min was determined using the double layer method. The experiment was repeated three times. Finally, the burst size was calculated as the ratio of the mean yield of phage particles liberated to the initial count of infected bacterial cells at the beginning of the latent period [34].

### 2.7. Temperature, pH, and Chloroform Stability

For the temperature stability test, *Aeromonas* phage Ahy-yong1 suspensions (3.18 × 10^10^ PFU/mL) were incubated at 30, 40, 50, 60, and 70 °C, respectively, and the phage titers at 30, 60, 90, and 120 min was analyzed. For the pH stability assessment, *Aeromonas* phage Ahy-yong1 suspensions (3.18 × 10^10^ PFU/mL) were adjusted to pH 2, 3, 4, 5, 6, 7, 8, 9, 10, 11, and 12 using NaOH or HCl, incubated at 29 °C for 2 h, and the phage titers were analyzed. For testing the sensitivity of *Aeromonas* phage Ahy-yong1 to chloroform, the phage suspensions (3.18 × 10^10^ PFU/mL) were added with chloroform to 1% and 5% (*V/V*), respectively, sufficiently mixed, and incubated at 29 °C for 2 h. The same volume of PBS (0.01 M) was added instead of chloroform in the control groups. Phage titers were analyzed by using the double-layer agar method. The above experiments were repeated three times.

### 2.8. The Ability of A. hydrophila A18 to Form Biofilm and the Biofilm-Removal Ability of Aeromonas Phage Ahy-Yong1

A biofilm assay was used to test the ability of *A. hydrophila* A18 to form biofilm as described [35]. *A. hydrophila* A18 was grown in TSB medium at 29 °C with shaking at 180 rpm for 16 h. Then, 100 µL of the fresh cultures were diluted with 20 mL TSB medium. Respectively, the diluted cultures and TSB medium were aliquoted into a 96-well microtiter plate in triplicate (200 µL/well) and incubated at 29 °C for 24 h. After three washes with PBS, the plate was dried at room temperature for 60 min. Following that, 200 µL of methyl alcohol was added into each well and maintained at room temperature for 30 min. Then, methyl alcohol was removed. Each well was filled with 200 µL of 1% (*w/v*) crystal violet solution. The staining time was 30 min. After removing the crystal violet solution, the wells were gently washed with sterile deionized water. After drying, 200 µL of 33% (*V/V*) glacial acetic acid was added into each well. The amounts of biofilm were indirectly determined by measuring the OD_590_ values of the crystal violet-stained biofilm and cells with a microplate reader. The experiment was repeated three times.

To test the biofilm-removal ability of *Aeromonas* phage Ahy-yong1, the following experiments were carried out. First, 200 µL of the diluted *A. hydrophila* A18 cultures described above were added to each well of a 96-well microtiter plate and incubated at 29 °C for 24 h. Respectively, 200 µL of *Aeromonas* phage Ahy-yong1 suspensions (2.35 × 10^10^ PFU/mL) and PBS (as control) was added into each well in triplicate and incubated at 29 °C for 2 h. The subsequent operations including methyl alcohol treatment, crystal violet staining, and biofilm quantification were carried out in the way described in the previous paragraph. The experiment was repeated three times.

### 2.9. Genome Sequencing and Analysis

The phage Ahy-yong1 suspensions were centrifuged for 20 min at 10,000× *g*, filtered through a 0.22 µm nitrocellulose filter, and pretreated with DNase and RNase. The phage genome was extracted from the filtrate using the High Pure Viral kit (Roche, Product No.: 11858882001, Basel, Switzerland). The genomic library was constructed with the NEBNextUltra^TM^ II DNA Library Prep Kit (#E7645) (NEB, Beijing, China) for Illumina. Genome sequencing was performed using the Illumina MiSeq (San Diego, CA, USA) sequencer to obtain 2 × 300 bp paired-end reads. Trimmomatic-0.36 was used for filtering out the low-quality (Q-value < 20) reads and adapter sequences. Assembling the clean reads was performed using SPAdes 3.13.0 software (http://cab.spbu.ru/software/spades/, accessed on 26 August 2021). Phage termini were predicted by using the software PhageTerm [36] and the reported method [37]. tRNA was predicted with tRNAscan-SE software (https://lowelab.ucsc.edu/tRNAscan-SE/, accessed on 10 February 2022) [38]. The antibiotic resistance and virulence genes were searched in the comprehensive antibiotic resistance database (CARD) (http://arpcard.mcmaster.ca, accessed on 12 February 2022) and the virulence factor database (VFDB) (http://www.mgc.ac.Cn/VFs/main.htm, accessed on 12 February 2022), respectively. The promoter was predicted using the Softberry server (http://www.softberry.com/berry.phtml, accessed on 18 February 2022). ORFs were predicted and annotated by the RAST website server (https://rast.nmpdr.org/rast.cgi, accessed on 18 August 2021) [39] and verified with BLASTp (https://blast.ncbi.nlm.nih.gov/Blast.cgi, accessed on 17 February 2022) (database: non-redundant protein sequences; *E*-value ≤ 10^−5^; percentage identity between the aligned sequences, >45%), HMMER (https://toolkit.tuebingen.mpg.de/tools/hhpred, accessed on 3 March 2022) [40] (database: Pfam, TIGRFAM, Gene3D, Superfamily, PIRSF and TreeFam; *E*-value ≤ 10^−5^), and HHpred server (https://www.ebi.ac.uk/Tools/hmmer/search/hmmscan, accessed on 6 March 2022) [41] (database: PDB_mmCIF70_31_Jul, PDB_mmCIF30_31_Jul, Pfam-A_v35 and NCBI_Conserved_Domains (CD)_v3.19; *E*-value ≤ 10^−5^; percentage possibility of homologous sequences > 96%).

### 2.10. Taxonomic Analysis

Nucleotide sequence comparisons were primarily made using BLASTn against standard databases (Nucleotide collection (nr/nt)). A genome comparison map of *Aeromonas* phage Ahy-yong1 and the closest related phages (*Aeromonas* phage LAh1, CF7 and Aph1) sharing the highest identity with Ahy-yong1 in a BLASTn search was made by a genome comparison visualizer [42]. The average nucleotide identity (ANI) values between *Aeromonas* phage Ahy-yong1 and the most relative phages were calculated using the EzGenome web server (http://www.ezbiocloud.net/ezgenome/ani, accessed on 11 February 2022) [43]. The in silico DNA–DNA hybridization (isDDH) identities between *Aeromonas* phage Ahy-yong1 and the most relative phages were calculated using GGDC web server (http://ggdc.dsmz.de, accessed on 11 February 2022) [44]. The pairwise sequence comparison (PASC) tool (http://www.ncbi.nlm.nih.gov/sutils/pasc/, accessed on 7 May 2022) [45] was used to calculate nucleotide sequence similarity between *Aeromonas* phage Ahy-yong1 and other phages in current databases. The virus intergenomic distance calculator (VIRIDIC) server (http://rhea.icbm.uni-oldenburg.de/VIRIDIC/, accessed on 7 May 2022) [46] was used for calculating phage intergenomic similarities (BLASTn parameters, ‘-word_size 7-reward 2-penalty-3-gapopen 5-gapextend 2’). The ViPTree server (https://www.genome.jp/viptree/, accessed on 10 May 2022) [47] was used to generate a proteomic tree based on genome wide sequence similarities, computed by tBLASTx. An original proteomic tree was constructed with all the reference genomes of 6,306 viruses in the databases. Sixty-two classified phages of *Caudoviricetes* class with shorter evolutionary distance from Ahy-yong1 in the original tree and one unclassified phage (*Aeromonas* phage LAh1) sharing the highest identity with Ahy-yong1 in BLASTn searchwere applied to construct a detailed proteomic tree.

### 2.11. Protective Effects of Aeromonas Phage Ahy-Yong1 against A. hydrophila in Brocade Carp (Cyprinus aka Koi)

The protective experiment of *Aeromonas* phage Ahy-yong1 against *A. hydrophila* in brocade carp was performed at 25 °C with continuous aeration. First, 300 healthy brocade carps with length of 7–8 cm were randomly divided into five groups. Each fish of the blank group was successively injected intraperitoneally twice with 0.01 M PBS (Table 2). Each fish of the control group was successively injected intraperitoneally with *A. hydrophila* A18 (10^8^ CFU/mL) and 0.01 M PBS. Each fish of the test group I was successively injected intraperitoneally with *A. hydrophila* A18 (10^8^ CFU/mL) and *Aeromonas* phage Ahy-yong1 (10^7^ PFU/mL). Each fish of the test group II was successively injected intraperitoneally with *Aeromonas* phage Ahy-yong1 (10^7^ PFU/mL) and *A. hydrophila* A18 (10^8^ CFU/mL). The injection time intervals were 2 hours and the injection volume was 100 µL. Each fish of the test group III was injected with 100 µL of *Aeromonas* phage Ahy-yong1 (10^7^ PFU/mL) immediately after the injection of 100 µL of *A. hydrophila* A18 (10^8^ CFU/mL). The fish mortality of each group was recorded daily. Each group was performed in triplicate. The relative protection rate was calculated by the ratio of the difference between average mortality of the control groups and average mortality of the test groups to average mortality of the control groups.

To analyze the in vivo dynamics of phage titer and host cell number, three additional test groups (I, II, and III) and one control group were set with the same treatment as above. At different time points (0 h, 2 h, 4 h, 6 h, 8 h, 12 h, 24 h, 48 h, 72 h, 96 h, and 120 h) post the injection of *Aeromonas* phage Ahy-yong1, 3 brocade carp samples from each group were collected. The surface of the collected fish samples were wiped with alcohol cotton balls in a super-clean worktable. Fish samples were dissected under aseptic condition. First, 0.06 g of muscle of each sampled brocade carp were cut off and mashed. Then, 1 mL of 0.01 M PBS was added to the mashed muscle and mixed well in an Eppendorf tube. The phage titers in the mixtures were analyzed by the double-layer agar method. The cell numbers in the mixtures were monitored by plate counting method.

### 2.12. Statistical Analysis

All statistical analyses were performed using SPSS. Results were expressed as means ± standard deviation (SD). One-way ANOVA was used to determine the statistical significance, and the significance level was defined as * *p* ≤ 0.05, ** *p* ≤ 0.01 and *** *p* ≤ 0.001.

## 3. Results

### 3.1. Antibiotic Susceptibility of A. hydrophila A18

*A. hydrophila* A18 was resistant to 6 of 16 tested antibiotics, which were cephalexin, penicillin, amoxicillin, azithromycin, clindamycin, and vancomycin (Figure S1, Table S1). Its sensitivity was intermediate to tetracycline, rifampicin, and doxycycline. The resistance is related to the type of the antibiotics. For example, *A. hydrophila* A18 was resistant to penicillin and amoxicillin, which belong to penicillins; *A. hydrophila* A18 was moderately resistant totetracycline and doxycycline, which belong to tetracyclines; *A. hydrophila* A18 was sensitive to kanamycin, gentamicin, and tobramycin, which belong to aminoglycosides. Multiple-drug-resistant (MDR) was defined as resistance to three or more antimicrobials or antimicrobial groups [48], *A. hydrophila* A18, isolated from an aquatic product market, was classified as MDR as it exhibited resistance to three classes of antibiotics. As stated in the introduction, the rising drug resistance in aquatic bacteria promotes aquatic environments to act as a gateway for antimicrobial resistance. Here, we call for more attention to the prevention and control of aquatic pathogenic bacteria.

### 3.2. Plaque and Phage Morphology

**Figure 1:**
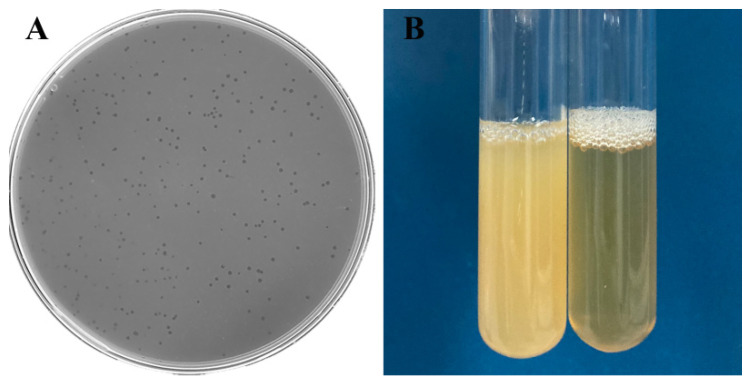
Visualization of Ahy-yong1 plaques and *Aeromonas hydrophila* A18 cultures. (**A**) *Aeromonas* phage Ahy-yong1 formed clear and circular plaques on *A. hydrophila* A18 lawns. Small plaques can be seen in 3 h, and the average diameter of the plaques reached 0.84 mm in 4 h. (**B**) Normal *A. hydrophila* A18 culture (left) and lysateofphageAhy-yong1-infected *A. hydrophila* A18 (right).

**Figure 2:**
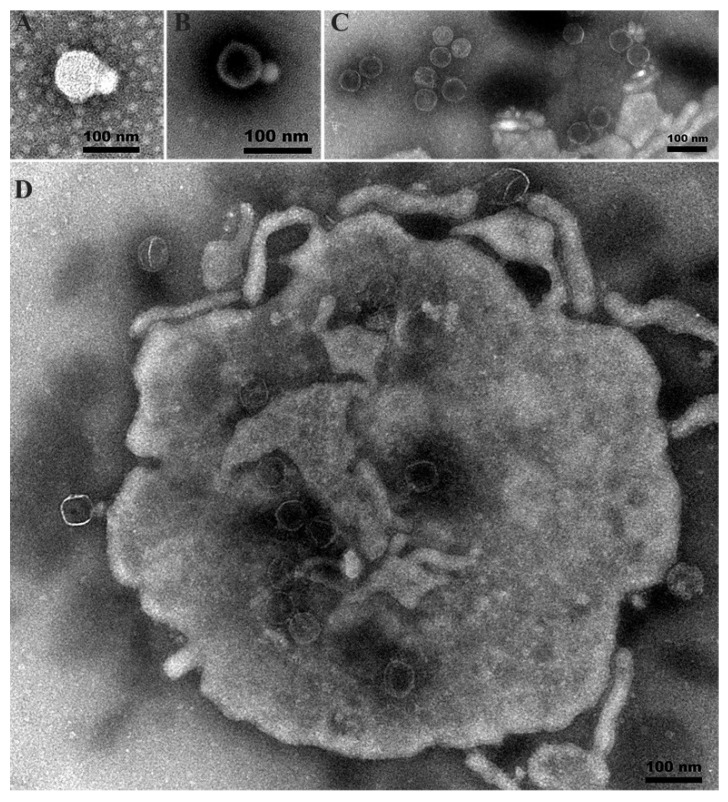
Transmission electron microscopy of negatively stained *Aeromonas* phage Ahy-yong1 and *A. hydrophila* A18 cell infected with Ahy-yong1. (**A**–**C**) Free intact-matureAhy-yong1 virion. Ahy-yong1 possesses a head with a diameter of 66 nm and a short non-contractile tail of 26 nm in length and 32 nm in width under a transmission electron microscope. (**D**) Immature phage particles being packaged within a viral factory in an infected *A. hydrophila* A18 cell.

On *A. hydrophila* lawns, *Aeromonas* phage Ahy-yong1 formed clear, discrete, circular plaques of 0.84 mm average diameter in 4 h (Figure 1A). Addition of a single plaque to broth cultures demonstrated evidence of clearing (Figure 1B). Ahy-yong1 possesses a small head with a diameter of approximately 66 nm and a short non-contractile tail of approximately 26 nm in length and 32 nm in width under a transmission electron microscope (Figure 2A–C). Phage particles being packaged within viral factories in infected *A. hydrophila* A18 cells were observed (Figure 2D).

### 3.3. Host Range

Ahy-yong1 in the three dilutions tested all formed transparent spots on the lawn of the indicator bacterium (*A. hydrophila* A18). Among the 35 tested bacterial strains, only the indicator bacterium lawns displayed transparent zones (Table 1). Among the tested strains, there were three other *A. hydrophila* strains, in addition to A18. *A. hydrophila* ATCC49140 is a clinical isolate and ATCC bacterial type strain from USA. *A. hydrophila* Ah2 was isolated by Zhu et al. from the turtle farm at Haining County of Zhejiang Province, China [49]. *A. hydrophila* AS1.1801 was obtained from Microbiological Culture Collection Center of Guangdong Institute of Microbiology. The results indicated that phage Ahy-yong1 has strict host specificity (strain specific).

### 3.4. Optimal Multiplicity of Infection (MOI), Optimal Adsorption Time, and One-Step Growth Curve

After 4 h of incubation, all the cultures became clear. Phage titers in the lysates under MOIs of 0.1, 0.01, 0.001, and 0.0001 were 5.7 × 10^8^ ± 2.6 × 10^7^, 1.1 × 10^8^ ± 1.7 × 10^7^, 1.6 × 10^8^ ± 1.2 × 10^7^, 3.1 × 10^8^ ± 1.4 × 10^7^ PFU/mL, respectively. MOI at 0.1 was proposed as optimal.

The adsorption of Ahy-yong1 to *A. hydrophila* A18 is very efficient. The adsorption rate can reach 99.75 ± 0.03% in 2 min at 29 °C (Figure S2).

**Figure 3:**
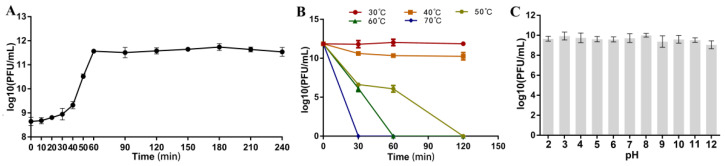
The one-step growth curve under the MOI of 0.1 and the results of the temperature stability and pH stability test of *Aeromonas* phage Ahy-yong1. (**A**) The one-step growth curve demonstrated that the latent period of *Aeromonas* phage Ahy-yong1 was 10 min, followed by a burst period of 50 min. (**B**) *Aeromonas* phage Ahy-yong1 was very stable at 30 °C, maintaining constant production for over 120 min; relatively stable at 40 °C; not stable at 50 °C, 60 °C, and 70 °C. Phage Ahy-yong1 eventually became inactive when the temperature exceeded 50 °C. (**C**) *Aeromonas* phage Ahy-yong1 was found to be stable at pH 2 to 12, indicating that even extremes of pH could not affect infectivity of phage Ahy-yong1. All values represent the mean of triplicate measurements, and error bars represent the standard deviations (*n* = 3).

The one-step growth curve at MOI of 0.1 (Figure 3A) demonstrated that the latent period of *Aeromonas* phage Ahy-yong1 was approximately 10 min, followed by a burst period of 50 min. The burst size of phage Ahy-yong1 at MOI of 0.1 was 637 PFU/cell, which was calculated as the ratio of mean yield of phage particles liberated to the mean phage particles that infected the bacteria in the initial latent period.

### 3.5. Temperature, pH, and Chloroform Stability

Results of the thermal stability test showed that *Aeromonas* phage Ahy-yong1 was very stable at 30 °C, maintaining constant titer; relatively stable at 40 °C with a slight decline of titer at the beginning and then maintaining constant titer; not stable at 50 °C, 60 °C, and 70 °C (Figure 3B). The phage completely inactivated at 50 °C, 60 °C, and 70 °C in 120, 60, and 30 min respectively. As shown in Figure 3C, *Aeromonas* phage Ahy-yong1 was found to be stable at pH 2 to 12, indicating that Ahy-yong1 is stable to acid and alkali. The average phage titers of the test groups treated with 1% and 5% chloroform were 1.33 × 10^10^ and 4.25 × 10^9^ PFU/mL, respectively, which were not much lower than the untreated control group (3.18 × 10^10^ PFU/mL). Therefore, *Aeromonas* phage Ahy-yong1 is not significantly sensitive to chloroform.

### 3.6. The Ability of A. hydrophila A18 to Form Biofilm and the Biofilm-Removal Ability of Aeromonas Phage Ahy-Yong1

**Figure 4:**
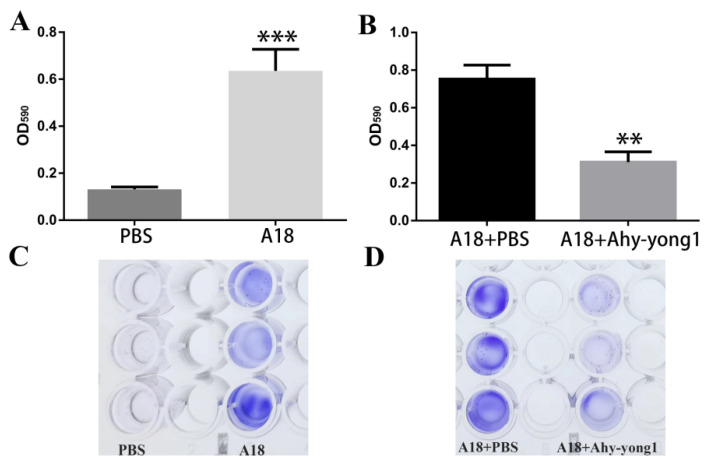
The ability of *A. hydrophila* A18 to form biofilms and the ability of *Aeromonas* phage Ahy-yong1 to eliminate biofilm. (**A**,**B**) The OD_590_ of values of the crystal-violet-stained biofilm and cells. (**C**,**D**) The wells stained with crystal violet. All values represent the mean of triplicate measurements, and error bars represent the standard deviations (*n* = 3). ** *p* < 0.01 and *** *p* < 0.001.

The average OD_590_ values of the wells containing *A. hydrophila* A18 was 4.86 times of that of TSB medium (Figure 4A). *A. hydrophila* A18 revealed a strong ability to form biofilm. The average OD_590_ values of the wells containing *A. hydrophila* A18 treated with PBS was 2.42 times that of the wells containing *A. hydrophila* A18 treated with *Aeromonas* phage Ahy-yong1 (Figure 4B). The results indicated that *Aeromonas* phage Ahy-yong1 could effectively eliminate the biofilm formed by *A. hydrophila* A18.

### 3.7. Genome Analysis

**Figure 5:**
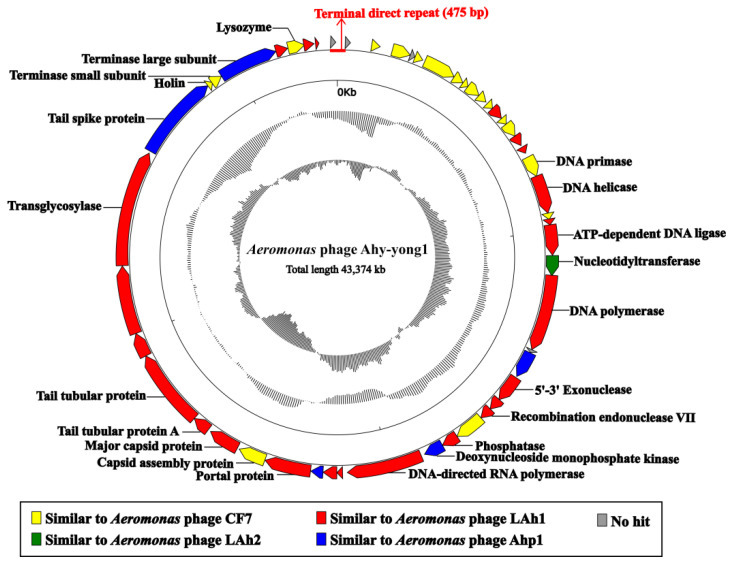
Genomic map of *Aeromonas* phage Ahy-yong1. From outside to inside, circle 1 shows the 52 predicted ORFs, circle 2 shows the size in pairs (kb), circle 3 displays the GC of the genome, and the innermost circle shows the GC skew plot (G − C)/(G + C). The direction of the arrow indicates the transcription direction of each gene. The color of each gene refers to the most similar phages, yellow stands for the gene similar to the *Aeromonas* phage and gray for the gene without similarity in the NCBI database.

Complete genome sequencing was conducted using high-throughput sequencing with an average sequencing depth of 1111-fold. *Aeromonas* phage Ahy-yong1 has a 43,374 bp double stranded DNA genome with a G+C content of 59.4%. The genomic termini were predicted based on NGS data by using the software PhageTerm [36] and identified according to the description [37]. The approach is based on the calculation and interpretation of three specific ratios, R1, R2, and R3, as suggested by Li et al. [37]. The result indicated that *Aeromonas* phage Ahy-yong1 has fixed termini with R1 = 235 > 100, R2 = 3, and R3 = 27. The genome of *Aeromonas* phage Ahy-yong1 contains terminal direct repeats of 475 bp (Figure 5). No tRNA gene was predicted in the genome. The absence of gene products predicted to be associated with virulence, antibiotic resistance, or a temperate lifestyle suggest that Ahy-yong1 could make a suitable candidate for biocontrol of *A. hydrophilia* in aquaculture. Twenty-two predicted promoters of the *Aeromonas* phage Ahy-yong1 genome was listed in Table S2. The genomic sequence of the *Aeromonas* phage Ahy-yong1 was submitted to the Genbank database under accession number OM654404.

The Ahy-yong1 genome contains 52 predicted open reading frames (ORFs), which are oriented in the same direction and cover 94.07% of the genome. Through the literature and genomic data search, we found that the closest relatives of Ahy-yong1 (several *Kayfunavirus* phages belong to the genus *Kayfunavirus* of the subfamily *Studiervirinae* of the family *Autographiviridae*) also have this characteristic, i.e., their ORFs were predicted to be transcribed from one DNA strand. The top hits in BLASTp searchers of all the 52 predicted ORFs are listed in Table S3. Forty-eight ORFs were most homologous with *Aeromonas* phages (identity: 46.04–100%), and the other four ORFs had no homology in current databases(https://blast.ncbi.nlm.nih.gov/Blast.cgi, accessed on 17 February 2022) (Table S3, Figure 5). The Ahy-yong1 genome revealed a mosaic organization. Its 24 ORFs were most homologous with *Aeromonas* phage LAh1; 18 ORFs were most homologous with *Aeromonas* phage CF7; 5 ORFs were most homologous with *Aeromonas* phage Ahp1; and 1 ORF was most homologous with *Aeromonas* phage LAh2.

**Figure 6:**
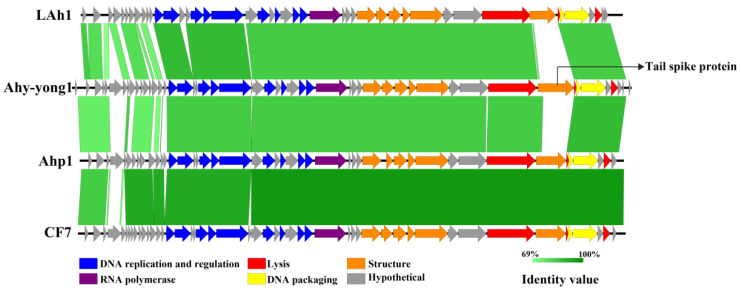
Genome comparison of the *Aeromonas* phage Ahy-yong1 and the three closest relatives (*Aeromonas* phage LAh1, *Aeromonas* phage CF7, and *Aeromonas* phage Ahp1). The orientation of the arrows indicates the direction of gene transcription. The color of each arrow refers to the functional categories: blue indicates DNA replication and regulation; yellow indicates DNA packaging; red indicates lysis; orange indicates structure; purple indicates RNA polymerase; gray indicates hypothetical protein. The homologous regions are represented by green bars. Light green to dark green represent low to high homology between genes.

The predicted ORFs could be classified as five functional categories: DNA replication and regulation, structure, lysis, DNA packaging, and hypothetical protein. Genomes of *Aeromonas* phage Ahy-yong1 and its close relatives revealed a modular organization (Figure 6).

The ORFs associated with DNA replication and regulation mainly situated the upstream of the Ahy-yong1 genome, coding putative DNA primase (ORF 17), DNA helicase (ORF 18), ATP-dependent DNA ligase (ORF 21), nucleotidyl transferase (ORF 22), DNA polymerase (ORF 23), 5′-3′ exonuclease (ORF 26), recombination endonuclease VII (ORF 28), phosphatase (ORF 30), deoxynucleoside monophosphate kinase (ORF 31), and DNA-dependent RNA polymerase (ORF 32). Nucleotidyl transferase is a CCA-adding enzyme adding the sequence cytidine(C)-cytidine(C)-adenosine(A) to the 3’ end of tRNAs, which is essential to tRNA aminoacylation and correct tRNA positioning in the ribosome [50]. The transcriptions of most phages depend on RNA polymerases of the hosts [51]. The predicted *DNA-dependent RNA polymerase* gene (ORF 32) in the genome of *Aeromonas* phage Ahy-yong1 imply that Ahy-yong1 may be primarily dependent on its own RNA polymerase rather than the host’s RNA polymerase for transcription.

ORF 36, ORF 37, ORF 38, ORF 39, ORF 40, and ORF 44 are associated with phage structure. Double-stranded (ds) DNA phages package their genomes into procapsid through the portal, which also acts as an initiator of capsid assembly and is involved in tail assembly [52]. ORF 36 of Ahy-yong1 encodes putative portal protein. ORF 37 encodes putative capsid assembly protein, which mediates the assembly of phage capsid protein. ORF 38 encodes putative major capsid protein. ORF 39 and 40 encode tail tubular proteins. ORF 44 encodes a tail spike protein.

ORF 46 and 47 are associated with DNA packaging. They were predicted to encode terminase small subunits and large subunits, respectively.

The lysis cassette of *Aeromonas* phage Ahy-yong1 genome contains three genes involved in the degradation of the host cell wall. ORF 43 encodes a putative transglycosylase, ORF 45 encodes putative holin. ORF 49 encodes putative lysozyme. Phage lytic transglycosylase can degrade the cell wall by catalyzing the cleavage of the β-1,4-glycosidic bond between N-acetylmuramic acid and N-acetylglucosamine [53]. Phage holincan form transmembrane holes on the host cell membrane, which enable the lysozyme to gain access to and degrade the cell wall [54].

Genome comparisons of *Aeromonas* phage Ahy-yong1 and the three closest relatives (*Aeromonas* phage LAh1, *Aeromonas* phage CF7, and *Aeromonas* phage Ahp1) were shown in Figure 6. Forty-five ORFs of Ahy-yong1 share high homology with the genes of the relatives (percentage identities ranged from 77.77–89.29%). Ahy-yong1 shows a syntenic arrangement of homologous genes with its closest relatives. Although other structure proteins of these phages were conserved, the tail spike proteins were not conserved. Only a small part of (N-terminus) the tail spike protein of Ahy-yong1 shared significant similarity with that of the three closest relatives (query coverage as low as 17.65%). The rest of the portion (the middle part and C-terminus) of the tail spike protein of Ahy-yong1 shared extremely low identity (0%) with the closest relatives (Figure 6). The tail spike protein is one of the key determinants for host specificity [55], the lack of observed conservation between the tail spike proteins of these phages may have to do with the strict host specificity of the phages.

### 3.8. Taxonomic Analysis

Although 69 genomes of phages against *A. hydrophila* were reported in the NCBI database or literature (Table S4), the highest identity shared between Ahy-yong1 and the closest relative was only 85.33% (query coverage 85%) in BLASTn scanning. All the top 13 BLASTn hits having the highest coverage with *Aeromonas* phage Ahy-yong1 were *Aeromonas* phages. The coverage and identity shared by Ahy-yong1 and the top seven related *Aeromonas* phages were 81%–85% and 82.07%–85.33%, respectively. The top seven related *Aeromonas* phages were *Aeromonas* phage LAh1, CF7, LAh3, LAh4, LAh2, LAh5, and Aph1, respectively. As the ANI values, is DDH values and intergenomic similarities between the five *Aeromonas* phages (LAh1, LAh2, LAh3, LAh4 and LAh5) (Table S3) are higher than the threshold of ANI (95%), DDH (70%), and intergenomic similarities (95%) [56,57] to discriminate viral species, they may be considered the same species. Taxonomic information concerning them was not found in the classification system of the International Committee on Taxonomy of Viruses (ICTV). The other two *Aeromonas* phages (CF7 and Aph1) both belong to the genus *Ahphunavirus* of the subfamily *Melnykvirinae* of the family *Autographiviridae* according to the latest release of the ICTV [58].

**Figure 7:**
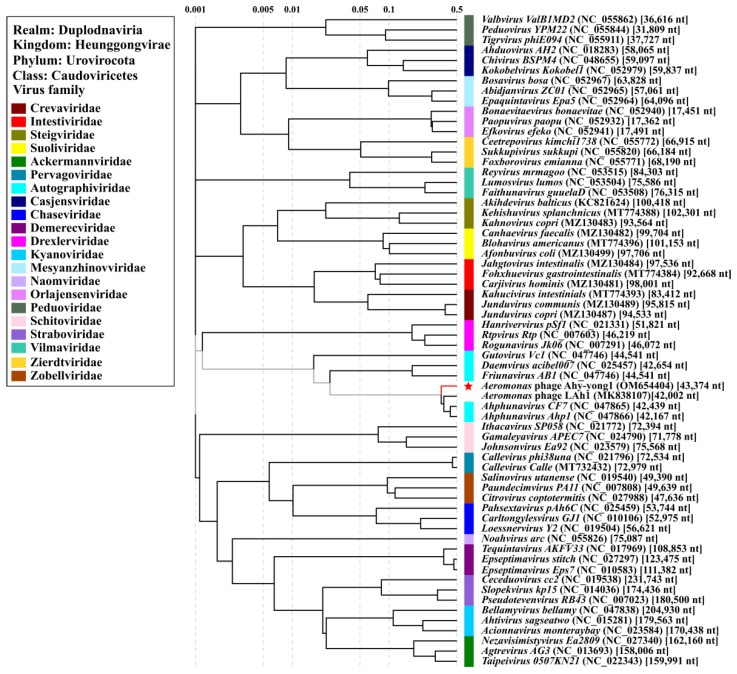
Proteomic tree based on the complete genome sequences of *Aeromonas* phage Ahy-yong1, 62 classified phages of *Caudoviricetes* class with shorter evolutionary distance from Ahy-yong1 in the original tree and unclassified *Aeromonas* phage LAh1 sharing the top highest homology with Ahy-yong1 in BLASTn scanning. Bacteriophage family assignments according to the official ICTV classification (March 2022) are provided with different color bars. The red star indicates phage Ahy-yong1. In the proteomic tree, *Aeromonas* phage Ahy-yong1 clustered with *Aeromonas* phages of the family *Autographiviridae*, especially closely related with *Aeromonas* phage LAh1, Ahp1, and CF7.

The ANI value and isDDH value between *Aeromonas* phage Ahy-yong1 and the seven most related *Aeromonas* phages ranged from 82.19% to 82.46% and 25.30% to 27.40% (Table S5), which were below the threshold values (95% of ANI and 70% of isDDH) to discriminate viral species. Results demonstrated Ahy-yong1 as a new species. In PASC scanning, the *Aeromonas* phage Ahy-yong1 shared the highest nucleotide sequence similarity of 75.63% with *Aeromonas* phage CF7. The intergenomic similarity between the two *Aeromonas* phages in VIRIDIC analysis was 75.2%. The Bacterial and Archaeal Virus Subcommittee (BAVS) of ICTV currently defined a new genus of viruses with the threshold values of nucleotide sequence similarity >70% [56,57]. Therefore, *Aeromonas* phage Ahy-yong1 and *Aeromonas* phage CF7 can be classified into the same genus. Moreover, in the detailed proteomic tree based on genome wide sequence similarities (Figure 7), *Aeromonas* phage Ahy-yong1 clustered with the phages of the family *Autographiviridae*, especially closely related with *Aeromonas* phage LAh1, CF7 and Ahp1 of *Ahphunavirus* genus. Hence, *Aeromonas* phage Ahy-yong1 was classified as a new species of *Ahphunavirus* genus in the subfamily *Melnykvirinae* of the family *Autographiviridae*.

### 3.9. Comparison of Phage Ahy-Yong1 and the Seven Most Related Aeromonas Phages

*Aeromonas* phage Ahy-yong1 shares the highest sequence similarity with seven *Aeromonas* phages including LAh1, CF7, LAh3, LAh4, LAh2, LAh5, and Ahp1. Information about CF7 is very limited. Due to the lack of literature, the comparison between Ahy-yong1 and the closest relatives is insufficient. However, from the known information, they share many similarities. The comparison of the characteristics among *Aeromonas* phage Ahy-yong1, LAh1, CF7, LAh3, LAh4, LAh2, LAh5, and Ahp1 are listed in Table S6 [59,60]. *Aeromonas* phage Ahy-yong1 and the top seven related *Aeromonas* phages share the following commonalities: strict host specificity, common genomic characteristics [59,60], and similar morphological characteristics. They obligately infect *A. hydrophila*. They all have double stranded DNA genomes of approximately 42,000 bp, with G+C contents of approximately 59%. Their gene arrangement and orientation are conserved. Their transcription directions of all the ORFs are all consistent. i.e., all genes in each genome are transcribed in the same direction. Their genomes all harbor a *DNA-dependent RNA polymerase* gene. Transmission electron microscopy revealed that Ahy-yong1, Ahp1, LAh1, LAh2, LAh3, LAh4, and LAh5 all exhibit a podo virus morphology. They all possess an icosahedral head (62–86 nm) and a short tail (8–26 nm) [59,60]. In addition, both Ahy-yong1 and LAh1 were found to be capable of disrupting *A. hydrophila* biofilm. The adsorption rate of Ahy-yong1 and Ahp1 both can reach >95% in 2 min, which indicated that adsorption of them to the host is very efficient. Nevertheless, Ahy-yong1 reveals its own unique characteristics. Ahy-yong1 exhibits morphological differences to its five relatives. The head diameter of the five relatives (LAh1, LAh2, LAh3, LAh4, LAh5) is 82 ± 4 nm, while that of Ahy-yong1 is approximately 66 nm, which is obviously smaller. The tail length of the five relatives is 8 ± 1 nm, and of Aph1 it is 12.5 nm, while that of Ahy-yong1 is approximately 26 nm, which is longer. On the phylogenetic tree (Figure 7), compared with its close relatives, Ahy-yong1 is relatively independent.

### 3.10. Protective Effects of Aeromonas Phage Ahy-Yong1 in Brocade Carp (Cyprinus aka Koi)

**Figure 8:**
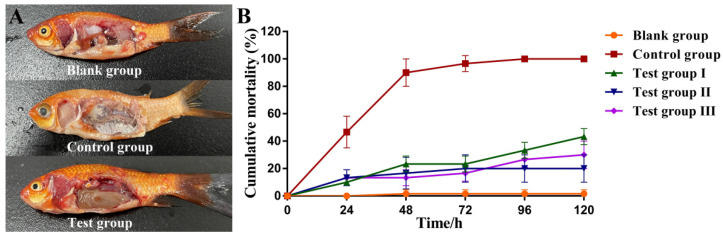
The internal organs and cumulative mortality curves of brocade carps (*Cyprinus aka* Koi) in the blank group, control group, and test groups. Each fish in the blank group was successively injected intraperitoneally twice with 0.01 M PBS. Each fish in the control group was successively injected intraperitoneally with *A. hydrophila* A18 (10^8^ CFU/mL) and 0.01 M PBS. Each fish in the test group I was successively injected intraperitoneally with *A. hydrophila* A18 (10^8^ CFU/mL) and *Aeromonas* phage Ahy-yong1 (10^7^ PFU/mL). Each fish in the test group II was successively injected intraperitoneally with *Aeromonas* phage Ahy-yong1 (10^7^ PFU/mL) and *A. hydrophila* A18 (10^8^ CFU/mL). The injection time intervals were 2 h and the injection volume was 100 µL. Each fish in the test group III was injected with 100 µL of *Aeromonas* phage Ahy-yong1 (10^7^ PFU/mL) immediately after the injection of 100 µL of *A. hydrophila* A18 (10^8^ CFU/mL). (**A**) The internal organs of brocade carps infected with *A. hydrophila* (control groups) were swollen and rotten and their eyes become cloudy relative to the blank groups and test groups. (**B**) The cumulative mortality of brocade carps in the blank groups was 1.7% ± 2.4%. The cumulative mortality of brocade carps in the test groups I, II, and III were 43.3% ± 4.7%, 20.0% ± 8.2%, and 30.0% ± 8.2%, respectively, which were significantly lower than those in the control groups, of which the cumulative mortality was 100.0%. All values represent the mean of triplicate measurements, and error bars represent the standard deviations (*n* = 3).

The pathological changes of the brocade carps in the negative control group (only be injected with *A. hydrophila*) were obvious. Their body color degenerated evidently, eyeballs became swollen and albino, and internal organs became swollen and rotten (Figure 8A). On the contrary, most brocade carps in the test groups (both treated with phage Ahy-yong1 and *A. hydrophila*) showed no obvious symptoms (Figure 8A).

The cumulative mortality of brocade carps in the blank groups was 1.7 ± 2.4% (Figure 8B, Table 2). The cumulative mortality of the negative control groups was 100.00%. The cumulative mortality of brocade carps in the test groups I, II, and III were 43.4 ± 4.7%, 20.0 ± 8.2%, and 30.0 ± 8.2%, respectively, which were significantly lower than those in the control groups (Figure 8B, Table 2). The relative protection rates of the test groups I, II, and III were 56.7 ± 4.7%, 80.0 ± 8.2%, and 70.0 ± 8.2%, respectively. The protection effect of the treatment mode of giving phage first and then bacteria is the best, followed by the simultaneous treatment. Results demonstrated that prevention was more effective than rescue.

**Figure 9:**
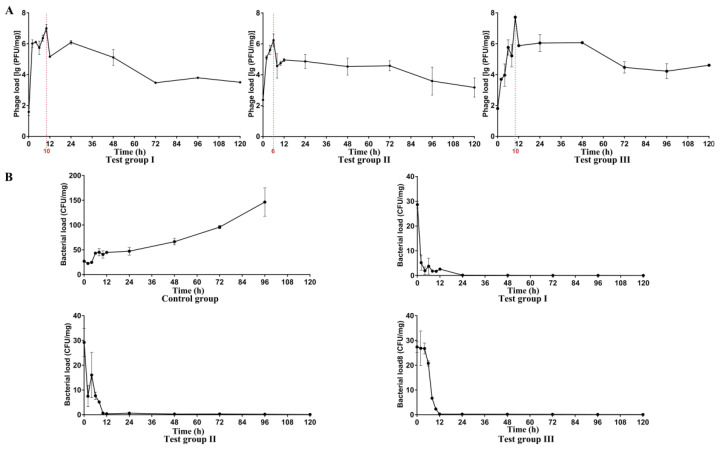
The dynamic curves of phage load and *A. hydrophila* A18 load in the muscles of the brocade carps. (**A**) The dynamic curves of phage load in the muscles of the brocade carps. (**B**) The dynamic curves of *A. hydrophila* A18 load in the muscles of the brocade carps. All values represent the mean of triplicate measurements, and error bars represent the standard deviations (*n* = 3).

The dynamic curves of phage load in the muscle of brocade carps (Figure 9A) showed that the change in trend of phage load was roughly coincident in the test I, II, and III groups, i.e., quickly peaked in 6 or 10 h, then gradually deceased, but maintained for a long time (at least 120 hours) at about twice of the load at 0 h (Figure 9A). Generally, the half-life of antibiotics was only a few hours. Ahy-yong1 remained active for up to 5 days in the muscle of brocade carps, which showed that *Aeromonas* phage Ahy-yong1 had a significant advantage over the antibiotics. The dynamic curves of *A. hydrophila* load in the muscle of brocade carps of the test groups all decreased quickly, with the *A. hydrophila* loads wall less than 1 CFU/mg in 96 h (Figure 9B). On the contrary, after a short slight decline, that in the control groups increased continuously on a large scale, reaching 147 CFU/mg at 96 h (Figure 9B).The result indicated that application of Ahy-yong1 by injection was able to reduce mortality caused by *A. hydrophila* A18 in vivo.

## 4. Discussion

*A. hydrophila* is an important opportunistic pathogen in various animals, including fish, causing ulcers, hemorrhages, furunculosis, and septicemia [12,13,14]. Antibiotics are used frequently and massively in aquaculture, which leads to antibiotic residues in aquatic products and induces multidrug-resistant bacteria. This results in pollution of the aquaculture environment and threatening human’s health. In this study, the antibiotic susceptibility test demonstrated the multiple antibiotic resistance of *A. hydrophila* A18. Artificial infection with *A. hydrophila* A18 resulted in 100% mortality in brocade carp. The infected brocade carp’ eyeballs became swollen and albino. Their body color degenerated evidently. *A. hydrophila* A18 can cause the internal organs of brocade carp to swell and rot.

Phage therapy is considered a potential method to treat bacterial infections. Studies on the isolation, identification, and therapy test of bacteriophages are necessary.

In this study, a lytic phage, *Aeromonas* phage Ahy-yong1, was isolated with *A. hydrophila* A18 and classified as a new species of *Ahphunavirus* genus in the subfamily *Melnykvirinae* of the family *Autographiviridae*. In 2019, ICTV removed the subfamily *Autographiviridae* and *Autographiviridae*-like viruses from the family *Podoviridae* of order *Caudovirales* and assigned a family rank, “*Autographiviridae*”, directly under the order *Caudovirales* [58]. In March this year, ICTV abolished the taxonomic unit of order *Caudovirales* and assigned the family *Autographiviridae* directly under the class *Caudoviricetes*. The dsDNA genomes of *Autographiviridae* phages are approximately 41 kb with conservative gene arrangement, comprising specific lysis cassettes and all encode a large (>100 kDa) single subunit RNA polymerase [58]. The defining characteristic of the family was the presence of the virion-encoded RNA polymerase from which the family derived its name; “Auto” and “Graphein” derived from the Greek meaning “self-writing” or “self-transcribing” [61]. The common morphological characteristic of *Autographiviridae* phages is that they all possess a small icosahedral head attached to a short tail. Ahy-yong1 also has these characteristics, i.e., harboring a 43,374 bp double stranded DNA genome with an RNA polymerase gene and possessing a small icosahedral head attached to a short tail.

In this study, a viral proteomic tree based on genome-wide similarities, instead of the routine phylogenetic tree based on sequence similarity of one or few genes, is used to gain quick insights into the classification of the target viral genome. Viral proteomic tree analysis was recommended by ICTV. The advantages of the genome-based proteomic trees are as follows: (i) highly diverse viral genomes can be analyzed together, (ii) no conserved gene in the genome is needed, (iii) it is likely less sensitive to genome rearrangement [47]. In this study, the result of the proteomic tree was confirmed by pair-wise ANI, isDDH, PASC, and VIRIDIC analysis. These were also conformed to its morphological characteristics.

*Aeromonas* phage Ahy-yong1 has very strict host specificity. Among the 35 tested bacterial strains, it only infected and lysed *A. hydrophila* A18. Most of the reported phages have a narrow host range (strainor species specific).The host range is likely bounded by the phylogenetic, geographical, and ecological distance between a phage and its potential hosts. The depth and breadth of bacterial and viral diversity, and difficulties in clearly delineating phage and bacterial taxonomy, make the formulation of specific definitions problematic [62]. The mechanism of the strict host-specificities of Ahy-yong1 needs further study. Phage host-specificity is a double-edged sword. On the one hand, narrow-host-range phages are less likely to disrupt the natural microbial flora and lower the probability of developing community-wide resistance. On the other hand, various phages may need to be isolated for each target strain [62]. Based on the accumulation of various phage isolates, phage cocktails may be a potential solution.

Previously reported *Aeromonas* phages have a wide range of burst sizes (5 PFU/cell to 608 PFU/cell) [30]. The burst size of phage Ahy-yong1 at MOI of 0.1 were 637 PFU/cell, which is higher than other *Aeromonas* phages. Candidate phages used in phage therapy must be not only effective but also safe. The therapeutic value of a candidate phage is related to growth kinetics, stability, host range, genomic features, and so on [63]. The characterization and genomic analysis indicated that *Aeromonas* phage Ahy-yong1 is a good candidate for control of *A. hydrophila* A18. First, its latent period (10 min) is short, which contributes to more rapidly establishing multiple parallel infections at high host cell concentrations [64]. Second, the phage was stable over a range of temperatures (30 °C and 40 °C), pH (2–12), and in the presence of chloroform, which is an advantage for the application in complex environments. Third, adsorption of Ahy-yong1 to *A. hydrophila* A18 is very efficient. The adsorption rate can reach 99.75 ± 0.03% in 2 min. Fourth, *Aeromonas* phage Ahy-yong1 has strict host specificity. This makes it unlikely to destroy the normal flora in application. Fifth, Ahy-yong1 can effectively remove the biofilm formed by *A. hydrophila* A18. Biofilm removal capacity is beneficial to phage therapy. Sixth, no virulence or antibiotic resistance genes were found in its genome, which strengthens the safety of the application of the phage. In addition, no integrase, *plasmid-replication* gene, transposase, repressor, and genome attachment site were found in its genome. Last but not least, results of the protection experiments of *Aeromonas* phage Ahy-yong1 against *A. hydrophila* in brocade carp (*C. aka* Koi) demonstrated the therapeutic and protective properties of the phage.

*Aeromonas* phage Ahy-yong1 delayed the death and reduced the mortality of brocade carps infected with *A. hydrophila* via injection. The relative protection rates of *Aeromonas* phage Ahy-yong1 to brocade carps against *A. hydrophila* A18 in the test groups I (rescue treatment 2 h after infection), II (prophylactic treatment), and III (rescue treatment immediately after infection) were 56.7 ± 4.7%, 80.0 ± 8.2%, and 70.0 ± 8.2%, respectively. The mortality of the prophylactic group was less than half of the group treated 2 hours after infection. The mortality of the group treated immediately after infection was 50% lower than that of the group treated 2 hours after infection. Results indicated that the protective effects of the prophylactic were better than rescue post infection, and the earlier the use of the phage post infection, the better the therapeutic effects were. The dynamic curves of phage load in the muscle of brocade carps in test groups I, II, and III showed that the phage Ahy-yong1 can remain active for at least 120 h. The dynamic curves of *A. hydrophila* load in brocade carps in test groups I, II, and III showed that 120 h after the treatment of the phage Ahy-yong1, the load of *A. hydrophila* in the muscles was less than 1 CFU/mg, which was much lower than that of the control group (147 CFU/mg). These indicated that Ahy-yong1 can effectively inhibit *A. hydrophila* A18 in vivo.

Previously, Le et al. carried out an experiment to test whether *Aeromonas* phage Φ2 and Φ5 could be protective to striped catfishes (*Pangasianodon hypophthalmus*) against *A. hydrophila* N17 [23]. Although they did not conduct experiments on the effect of treatment time to the protective effect, they conducted experiments on the effect of treatment dose to the protective effect. Striped catfishes were intraperitoneally injected with *A. hydrophila* N17 and phage Φ2 plus Φ5 with the MOI of 100, 1, and 0.01 respectively. The cumulative mortality of the tested groups was up to 0%, 45%, and 68.33%, respectively, yet that of the control groups, only injected with *A. hydrophila* N17, were 81.7% [23]. The results of Le et al. demonstrated that a higher MOI brings lower cumulative mortality. In this study, Ahy-yong1 was demonstrated to be protective for brocade carps infected with *A. hydrophila* A18 at the MOI of 0.1. The relative protection rates of all the test groups were all higher than that of *Aeromonas* phage Φ2 plus Φ5 at a MOI of 0.1 (44.9%), revealing the high protection efficiency of Ahy-yong1. Protective experiments on *Aeromonas* phage Ahy-yong1 at higher MOIs against *A. hydrophila* A18 in brocade carp should be carried out in the future.

Janelidze et al. evaluated the therapeutic potential of *Aeromonas* phage AhMtk13a in zebrafish (*Danio rerio*) challenged with *A. hydrophila* GW3–10 via intraperitoneal injection and passive immersion in aquaria water [3]. In experimental series 1 (immersion test) with direct application of phage AhMtk13a to aquaria water at MOI 10, the cumulative mortality of the tested groups was 44% (group I: AhMtk13a was added to the water immediately after bacterial challenge), 62% (group II: AhMtk13a was added to the water 4 h post bacterial challenge), and 47% (group III: AhMtk13a was added to the water 30 min before bacterial challenge), respectively, which were all lower than the mortality of 78% in the control group challenged with *A. hydrophila* GW3-10 [3]. The relative protection rates of the 3 groups were 43.59%, 20.52%, and 39.74%, respectively, which was basically in line with the law of this study, described above, i.e., the effect of earlier phage medication is better. The relative protection rates of AhMtk13a are lower than that of Ahy-yong1, which reveals the high protect efficiency of Ahy-yong1 again. In experimental series 2 with triple application of AhMtk13a phage at MOI 100, the cumulative mortality was 15% in phage-treated group (phage treatment immediately after bacterial challenge), while that was 55% in the control group [3]. These results also were in line with the law, described above, i.e., higher MOI brings a lower cumulative mortality.

Considering the convenience of operation in aquaculture, oral or immersion administration may be a better choice. Therefore, in the future research, it is feasible to try to use these two kinds of administration methods in therapy experiments. Additionally, the protection experiments of the phage against *A. hydrophila* A18 infection in other types of animals, especially mammals and poultry, are also worth trying.

## 5. Conclusions

A lytic phage, Ahy-yong1, against multi-antibiotic-resistant pathogen *A. hydrophila* was isolated. Ahy-yong1 is morphologically *Podoviridae*-like. Ahy-yong1 harbors a dsDNA genome of 43,374 bp with a G+C content of 59.4% and 52 ORFs, which are oriented in the same direction and cover 94.07% of the genome. Bioinformatics analysis indicated Ahy-yong1 as a new species of the *Ahphunavirus* genus of the *Autographiviridae* family of the *Caudoviricetes* class. Ahy-yong1 is stable to acid and alkali, stable at 30 °C, and not significantly sensitive to chloroform. In vitro and in vivo experiments demonstrated a high antibacterial rate of Ahy-yong1 against *A. hydrophila*. A single bacteriophage has the disadvantage of a narrow antibacterial spectrum. Ahy-yong1 showed strict host specificity. The bacteriophage cocktail strategy has the characteristics of wider host range, which can overcome the shortcomings of the specificity of a single bacteriophage, and is the main development direction of bacteriophage therapy in the future. As more and more bacteriophages are isolated and identified, and the research on bacteriophages is deepened, a solid foundation will be laid for the extensive use of bacteriophages.

## Figures and Tables

**Table 1:** Host range of *Aeromonas* phage Ahy-yong1.

Strain	Infectivity
*Aeromonas hydrophila* A18	+
*Aeromonas hydrophila* Ah2	-
*Aeromonas hydrophila* ATCC49140	-
*Aeromonas hydrophila* AS1.1801	-
*Aeromonas sobria* ATCC43979	-
*Aeromonas salmonicida* CGMCC1.16015	-
*Aeromonas veronii* CGMCC 1.927	-
*Aeromonas veronii* AV4	-
*Aeromonas Veyron*	-
*Hafnia paralvei* LY-23	-
*Hafnia psychrotolerans* CGMCC 1.12806	-
*Hafnia alvei* CGMCC 1.2026	-
*Shigella dysenteriae*	-
*Salmonella paratyphi*	-
*Escherichia coli* DH5α	-
*Pseudomonas aeruginosa*	-
*Lactococcus garvieae*	-
*Shewanellaputrefaciens*	-
*Citrobacter freundii*	-
*Enterobacter cloacae*	-
*Enterobacter hormaechei* 1322	-
*Vibrio harveyis*	-
*Vibrio harveyi* 1–5	-
*Vibrio alginolyticuss*	-
*Vibrio alginolyticus* WY	-
*Vibrio alginolyticus* LDF	-
*Vibrio mediterranei* 117-T6	-
*Vibrio Parahemolyticus* MCCC 1A11655	-
*Vibrio anguillarum*	-
*Vibrio campbellii* MCCC 1A08161	-
*Edwardsiellatarda* ET	-
*Edwardsiellaictaluri* ATCC 33202	-
*Edwardsiellahoshinae* ATCC 33379	-
*Edwardsiella piscicida* MCCC 1K03230	-
*Edwardsiellatarda* MCCC235	-

+, positive with the appearance of plaques on the site of spot; -, negative without the appearance of plaque on the site of spot.

**Table 2:** Treatment and cumulative mortality of brocade carps of the five groups in the protective experiment.

Groups	Treatment	Mortality
Blank group	Successive injection (*n* = 2) with 100 µL of 0.01 M PBS (Injection time interval: 2 h)	1.7 ± 2.4%
Control group	Successive injection with 100 µL of *A. hydrophila* A18 (10^8^ CFU/mL) and 0.01 M PBS(Injection time interval: 2)	100.0%
Test group I	Successive injection with 100 µL of *A. hydrophila* A18 (10^8^ CFU/mL) and phage Ahy-yong1 (10^7^ PFU/mL) (Injection time interval: 2 h)	43.3 ± 4.7%
Test group II	Successive injection with 100 µL ofphage Ahy-yong1 (10^7^ PFU/mL) and *A. hydrophila* A18 (10^8^ CFU/mL) (Injection time interval: 2 h)	20.0 ± 8.2%
Test group III	Injected with 100 µL of phage Ahy-yong1 (10^7^ PFU/mL) immediately after the injection of 100 µL of *A. hydrophila* A18 (10^8^ CFU/mL)	30.0 ± 8.2%

## Supplementary Materials

The following supporting information can be downloaded at: https://www.mdpi.com/article/10.3390/v14112498/s1, Figure S1: Results of antibiotic susceptibility test of *A. hydrophila* A18; Figure S2:The adsorption kinetics of *Aeromonas* phage Ahy-yong1; Table S1: Antibiotic susceptibility of A. hydrophila A18; Table S2: Predicted promoters in *Aeromonas* phage Ahy-yong1 genome; Table S3: ORF analysis of the *Aeromonas* phage Ahy-yong1 genome; Table S4: Taxonomic status or morphological characteristic of the phages against *Aeromonas hydrophila*; Table S5: The ANI value, isDDH value and intergenomic similarities between *Aeromonas* phage Ahy-yong1 and the 7 most related *Aeromonas* phages.

## References

[1] Pérez-Sánchez T., Mora-Sánchez B., Balcázar J.L. (2018). Biological approaches for disease control in aquaculture: Advantages, limitations and challenges. Trends Microbiol..

[2] Cabello F.C., Godfrey H.P., Buschmann A.H., Dölz H.J. (2016). Aquaculture as yet another environmental gateway to the development and globalization of antimicrobial resistance. Lancet Infect. Dis..

[3] Janelidze N., Jaiani E., Didebulidze E., Kusradze I., Kotorashvili A., Chalidze K., Porchkhidze K., Khukhunashvili T., Tsertsvadze G., Jgenti D. (2022). Phenotypic and genetic characterization of *Aeromonas hydrophila* phage AhMtk13a and evaluation of its therapeutic potential on simulated *Aeromonas* infection in *Danio rerio*. Viruses.

[4] Liu D., Van Belleghem J.D., de Vries C.R., Burgener E., Chen Q., Manasherob R., Aronson J.R., Amanatullah D.F., Tamma P.D., Suh G.A. (2021). The safety and toxicity of phage therapy: A review of animal and clinical studies. Viruses.

[5] Donati V.L., Dalsgaard I., Sundell K., Castillo D., Er-Rafik M., Clark J., Wiklund T., Middelboe M., Madsen L. (2021). Phage-mediated control of *Flavobacterium psychrophilum* in aquaculture: In vivo experiments to compare delivery methods. Front. Microbiol..

[6] Dedrick R.M., Guerrero-Bustamante C.A., Garlena R.A., Russell D.A., Ford K., Harris K., Gilmour K.C., Soothill J., Jacobs-Sera D., Schooley R.T. (2019). Engineered bacteriophages for treatment of a patient with a disseminated drug-resistant *Mycobacterium abscessus*. Nat. Med..

[7] Cisek A.A., Dąbrowska I., Gregorczyk K.P., Wyżewski Z. (2017). Phage therapy in bacterial infections treatment: One hundred years after the discovery of bacteriophages. Curr. Microbiol..

[8] Wittebole X., De Roock S., Opal S.M. (2014). A historical overview of bacteriophage therapy as an alternative to antibiotics for the treatment of bacterial pathogens. Virulence.

[9] Fernández-Bravo A., Figueras M.J. (2020). An update on the genus *Aeromonas*: Taxonomy, epidemiology, and pathogenicity. Microorganisms.

[10] Janda J.M., Abbott S.L. (2010). The genus *Aeromonas*: Taxonomy, pathogenicity, and infection. Clin. Microbiol. Rev..

[11] Aly S.M. (2013). A Review of Fish Diseases in the Egyptian Aquaculture Sector: Working Report.

[12] Anjur N., Sabran S.F., Daud H.M., Othman N.Z. (2021). An update on the ornamental fish industry in Malaysia: *Aeromonas hydrophila*-associated disease and its treatment control. Vet. World.

[13] Zhang Q., Lin Y., Zhang T., Wu Y., Fang P., Wang S., Wu Z., Hao J., Li A. (2021). Etiological characteristics of “tail blister disease” of Australian redclaw crayfish (*Cheraxquadricarinatus*). J. Invertebr. Pathol..

[14] Wang J.L., Meng X.L., Lu R.H., Wu C., Luo Y.T., Yan X., Li X.J., Kong X.H., Ni G.X. (2015). Effects of *Rehmanniaglutinosa* on growth performance, immunological parameters and disease resistance to *Aeromonas hydrophila* in common carp (*Cyprinus carpio* L.). Aquaculture.

[15] Lee S.W., Wendy W. (2017). Antibiotic and heavy metal resistance of *Aeromonas hydrophila* and *Edwardsiella tarda* isolated from red hybrid tilapia (*Oreochromis* spp.) coinfected with motile aeromonas septicemia and edwardsiellosis. Vet. World.

[16] Vivekanandhan G., Savithamani K., Hatha A.A., Lakshmanaperumalsamy P. (2002). Antibiotic resistance of *Aeromonas hydrophila* isolated from marketed fish and prawn of South India. Int. J. Food Microbiol..

[17] Zhang Y., Jia K.X., Zhang X.W., Gao N., Liu Z.Q., Liu C.Y., Shan X.F., Qian A.D., Zhang X., Xu F.Y. (2021). Biological characteristics of phage PZL-Ah1 and its therapeutic effect on *Aeromonas hydrophila* infection. Chin. J. Prev. Vet. Med..

[18] Easwaran M., Dananjaya S.H.S., Park S.C., Lee J., Shin H.J., De Zoysa M. (2017). Characterization of bacteriophage pAh-1 and its protective effects on experimental infection of *Aeromonas hydrophila* in Zebrafish (*Danio rerio*). J. Fish Dis..

[19] Jun J.W., Kim J.H., Shin S.P., Han J.E., Chai J.Y., Park S.C. (2013). Protective effects of the *Aeromonas* phages pAh1-C and pAh6-C against mass mortality of the cyprinid loach (*Misgurnus anguillicaudatus*) caused by *Aeromonas hydrophila*. Aquaculture.

[20] Dien L.T., Ky L.B., Huy B.T., Mursalim M.F., Kayansamruaj P., Senapin S., Rodkhum C., Dong H.T. (2022). Characterization and protective effects of lytic bacteriophage pAh6.2TG against a pathogenic multidrug-resistant *Aeromonas hydrophila* in Nile tilapia (*Oreochromis niloticus*). Transbound. Emerg. Dis..

[21] Akmal M., Rahimi-Midani A., Hafeez-Ur-Rehman M., Hussain A., Choi T.J. (2020). Isolation, characterization, and application of a bacteriophage infecting the fish pathogen *Aeromonas hydrophila*. Pathogens.

[22] Tu V.Q., Nguyen T.T., Tran X.T.T., Millard A.D., Phan H.T., Le N.P., Dang O.T.H., Hoang H.A. (2020). Complete genome sequence of a novel lytic phage infecting *Aeromonas hydrophila*, an infectious agent in striped catfish (*Pangasianodon hypophthalmus*). Arch. Virol..

[23] Le T.S., Nguyen T.H., Vo H.P., Doan V.C., Nguyen H.L., Tran M.T., Tran T.T., Southgate P.C., Kurtböke D.I. (2018). Protective effects of bacteriophages against *Aeromonas hydrophila* species causing Motile *Aeromonas* Septicemia (MAS) in striped catfish. Antibiotics.

[24] Gao X.N., Li J.M., Sang R.X., Ding Q.H., Xu H.Y. (2021). Isolation, identification and biological characteristics of a bacteriophage against *Aeromonas hydrophila*. China Feed.

[25] Jia K.X. (2021). Isolation, Identification and Genomic Analysis of *Aeromonas hydrophila* Phage PZL-Ah152 and Functional Exploration of Depolymerase Dep47. Master’s Thesis.

[26] Huo S.T., Jiao H.Q., Li Q., Gu Z.M., Liu X.Q. (2021). Isolation, identification and preliminary application of *Aeromonas hydrophila* phage from *Procambarus clarkii*. Acta Hydrobiol. Sin..

[27] Lv S.J., Liu L., Cao Z., Lu S.J., Lin F. (2018). Isolation and functional identification of a phage against *Aeromonas hydrophila* isolated from *Trionyx sinensis*. Jiangsu Agric. Sci..

[28] Gao S.S. (2016). Isolation and Biological Characteristics Analysis of *Aeromonas hydrophila* Bacteriophages. Master’s Thesis.

[29] Shen C.J. (2013). Biological Characteristics and Genomic Analysis of *Aeromonas hydrophila* Bacteriophages. Master’s Thesis.

[30] Pereira C., Duarte J., Costa P., Braz M., Almeida A. (2022). Bacteriophages in the control of *Aeromonas* sp. in aquaculture systems: An integrative view. Antibiotics.

[31] Lin W., Li D.F., Gao M.M., Qin W.N., Xu L.H., Pan L.T., Liu W.C., Fan H., Mi Z.Q., Tong Y.G. (2021). Isolation, characterization and biocontrol efficacy of a T4-like phage virulent to multidrug-resistant *Enterobacter hormaechei*. Dis. Aquat. Organ..

[32] Ghosh K., Senevirathne A., Kang H.S., Hyun W.B., Kim J.E., Kim K. (2018). Complete nucleotide sequence analysis of a novel *Bacillus subtilis*-infecting bacteriophage BSP10 and its effect on poly-gamma-glutamic acid degradation. Viruses.

[33] Gutiérrez S., Pirolles E., Yvon M., Baecker V., Michalakis Y., Blanc S. (2015). The multiplicity of cellular infection changes depending on the route of cell infection in a plant virus. J. Virol..

[34] Walakira J.K., Carrias A.A., Hossain M.J., Jones E., Terhune J.S., Liles M.R. (2008). Identification and characterization of bacteriophages specific to the catfish pathogen, *Edwardsiellaictaluri*. J. Appl. Microbiol..

[35] Cramton S.E., Gerke C., Götz F. (2001). In vitro methods to study staphylococcal biofilm formation. Method Enzymol..

[36] Garneau J.R., Depardieu F., Fortier L.C., Bikard D., Monot M. (2017). PhageTerm: A tool for fast and accurate determination of phage termini and packaging mechanism using next-generation sequencing data. Sci. Rep..

[37] Li S., Fan H., An X., Fan H., Jiang H., Chen Y., Tong Y. (2014). Scrutinizing virus genome termini by high-throughput sequencing. PLoS ONE.

[38] Schattner P., Brooks A.N., Lowe T.M. (2005). The tRNAscan-SE, snoscan and snoGPS web servers for the detection of tRNAs and snoRNAs. Nucleic Acids Res..

[39] Aziz R.K., Bartels D., Best A.A., DeJongh M., Disz T., Edwards R.A., Formsma K., Gerdes S., Glass E.M., Kubal M. (2008). The RAST server: Rapid annotations using subsystems technology. BMC Genom..

[40] Potter S.C., Luciani A., Eddy S.R., Park Y., Lopez R., Finn R.D. (2018). HMMER web server: 2018 update. Nucleic Acids Res..

[41] Zimmermann L., Stephens A., Nam S.Z., Rau D., Kübler J., Lozajic M. (2018). A completely reimplemented MPI Bioinformatics Toolkit with a new HHpred server at its core. J. Mol. Biol..

[42] Sullivan M.J., Petty N.K., Beatson S.A. (2011). Easyfig: A genome comparison visualizer. Bioinformatics.

[43] Figueras M.J., Beaz-Hidalgo R., Hossain M.J., Liles M.R. (2014). Taxonomic affiliation of new genomes should be verified using average nucleotide identity and multilocus phylogenetic analysis. Genome Announc..

[44] Meier-Kolthoff J.P., Auch A.F., Klenk H., Göker M. (2013). Genome sequence-based species delimitation with confidence intervals and improved distance functions. BMC Bioinform..

[45] Bao Y., Chetvernin V., Tatusova T. (2014). Improvements to pairwise sequence comparison (PASC): A genome-based web tool for virus classification. Arch. Virol..

[46] Moraru C., Varsani A., Kropinski A.M. (2020). VIRIDIC-A novel tool to calculate the intergenomic similarities of prokaryote-infecting viruses. Viruses.

[47] Nishimura Y., Yoshida T., Kuronishi M., Uehara H., Ogata H., Goto S. (2017). ViPTree: The viral proteomic tree server. Bioinformatics.

[48] Magiorakos A.P., Srinivasan A., Carey R.B., Carmeli Y., Falagas M.E., Giske C.G., Harbarth S., Hindler J.F., Kahlmeter G., Olsson-Liljequist B. (2012). Multidrug-resistant, extensively drug-resistant and pandrug-resistant bacteria: An international expert proposal for interim standard definitions for acquired resistance. Clin. Microbiol. Infect..

[49] Zhu N.Y., Zheng X.Y., Cao F.F., Li Y.Y., Zheng T.L. (2017). Antimicrobial resistance of *Aeromonas hydrophila* isolated from soft-shelled turtle *Pelodiscus sinensis*. Acta Agric. Zhejiangensis.

[50] Kuhn C.D., Wilusz J.E., Zheng Y., Beal P.A., Joshua-Tor L. (2015). On-enzyme refolding permits small RNA and tRNA surveillance by the CCA-adding enzyme. Cell.

[51] Lavysh D., Sokolova M., Minakhin L., Yakunina M., Artamonova T., Kozyavkin S., Makarova K.S., Koonin E.V., Severinov K. (2016). The genome of AR9, a giant transducing *Bacillus* phage encoding two multisubunit RNA polymerases. Virology.

[52] Cuervo A., Fàbrega-Ferrer M., Machón C., Conesa J.J., Fernández F.J., Pérez-Luque R., Pérez-Ruiz M., Pous J., Vega M.C., Carrascosa J.L. (2019). Structures of T7 bacteriophage portal and tail suggest a viral DNA retention and ejection mechanism. Nat. Commun..

[53] Thunnissen A.M., Rozeboom H.J., Kalk K.H., Dijkstra B.W. (1995). Structure of the 70-kDa soluble lytic transglycosylase complexed with bulgecin A. Implications for the enzymatic mechanism. Biochemistry.

[54] Park T., Struck D.K., Deaton J.F., Young R. (2006). Topological dynamics of holins in programmed bacterial lysis. Proc. Natl. Acad. Sci. USA.

[55] Kunstmann S., Scheidt T., Buchwald S., Helm A., Mulard L.A., Fruth A., Barbirz S. (2018). Bacteriophage Sf6 tailspike protein for detection of *Shigella flexneri* pathogens. Viruses.

[56] Adriaenssens E., Brister J.R. (2017). How to name and classify your phage: An informal guide. Viruses.

[57] Adriaenssens E.M., Sullivan M.B., Knezevic P., van Zyl L.J., Sarkar B.L., Dutilh B.E. (2020). Taxonomy of prokaryotic viruses: 2018–2019 update from the ICTV Bacterial and Archaeal Viruses Subcommittee. Arch. Virol..

[58] Wang J.B., Lin N.T., Tseng Y.H., Weng S.F. (2016). Genomic characterization of the novel *Aeromonas hydrophila* phage Ahp1 suggests the derivation of a new subgroup from phiKMV-Like family. PLoS ONE.

[59] Kabwe M., Brown T., Speirs L., Ku H., Leach M., Chan H.T., Petrovski S., Lock P., Tucci J. (2020). Novel bacteriophages capable of disrupting biofilms from clinical strains of *Aeromonas hydrophila*. Front. Microbiol..

[60] Turner D., Kropinski A.M., Alfernas-Zerbini P., Buttimer C., Lavigne R., Bister J.R., Tolstoy I., Morozova V.V., Babkin I.V., Kozlova Y.N. Create One New Family (*Autographiviridae*) including Nine Subfamilies and 132 Genera in the Order *Caudovirales*. https://ictv.global/ICTV/proposals/2019.103B.zip.

[61] Lavigne R., Seto D., Mahadevan P., Ackermann H.W., Kropinski A.M. (2008). Unifying classical and molecular taxonomic classification: Analysis of the *Podoviridae* using BLASTP-based tools. Res. Microbiol..

[62] De Jonge P.A., Nobrega F.L., Brouns S.J.J., Dutilh B.E. (2018). Molecular and evolutionary determinants of bacteriophage host range. Trends Microbiol..

[63] Jacquemot L., Bettarel Y., Monjol J., Corre E., Halary S., Desnues C., Bouvier T., Ferrier-Pagès C., Baudoux A.C. (2018). Therapeutic potential of a new jumbo phage that infects *Vibrio coralliilyticus*, a widespread coral pathogen. Front. Microbiol..

[64] Abedon S.T. (1989). Selection for bacteriophage latent period length by bacterial density: A theoretical examination. Microb. Ecol..

