# Supplementary material for: Novel Aeromonas Phage Ahy-Yong1 and Its Protective Effects against Aeromonas hydrophila in Brocade Carp (Cyprinus aka Koi)
Source: Viruses. 2022 Nov 11;14(11):2498. doi: 10.3390/v14112498 (PMC9697113; doi:10.3390/v14112498)
Supplement: Supplementary file 1 [file viruses-14-02498-s001.zip › viruses-1845193-supplementary.pdf]

SUPPLEMENTARY MATERIALS

SUPPLEMENTARY FIGURES AND FIGURE LEGENDS

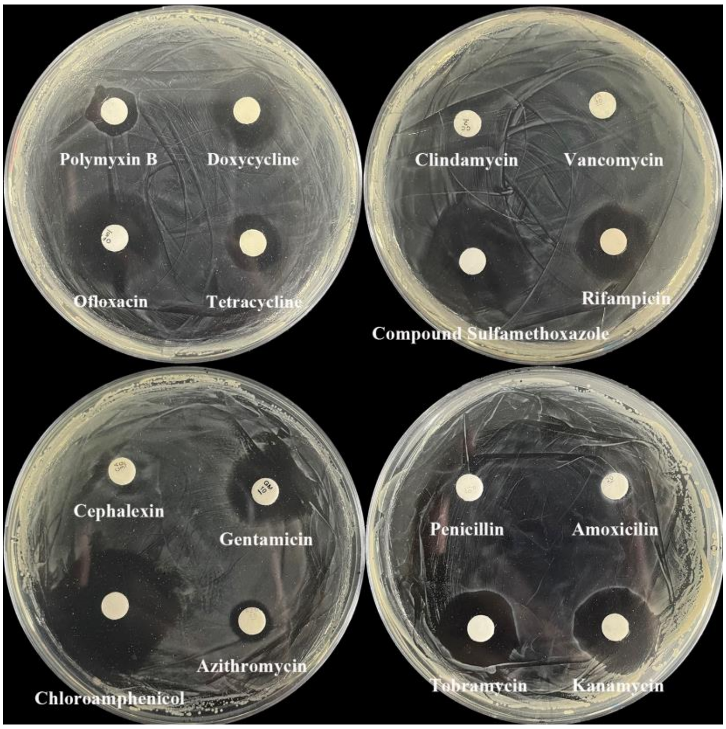

Figure S1. Results of antibiotic susceptibility test of *A. hydrophila* A18. The results showed that *A. hydrophila* A18 was resistant to 6 of 16 tested antibiotics, which were cephalexin, penicillin, amoxicillin, azithromycin, clindamycin, and vancomycin. Its sensitivity was intermediate to tetracycline, rifampicin and doxycycline.

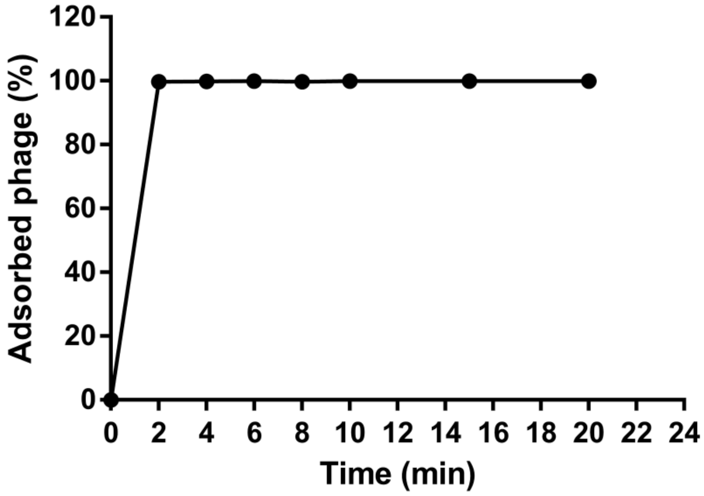

Figure S2. The adsorption kinetics of *Aeromonas* phage Ahy-yong1. Adsorption of Ahy-yong1 to *A. hydrophila* A18 is very efficient, and the adsorption rate can reach 99.75% ± 0.03% in 2 min at 29°C.

Table S1. Antibiotic susceptibility of *A. hydrophila* A18.

Tested antibiotics	Antimicrobial class	Inhibition zones	Sensitivity
Cephalexin	Cephems	7 mm	R
Penicillin	Penicillins	7 mm	R
Amoxicillin	Penicillins	7 mm	R
Azithromycin	Macrolides	10 mm	R
Clindamycin	Lincosamides	7 mm	R
Vancomycin	Glycopeptides	7 mm	R
Tetracycline	Tetracyclines	13 mm	I
Rifampicin	Ansamycins	17 mm	I
Doxycycline	Tetracyclines	15 mm	I
Kanamycin	Aminoglycosides	20 mm	S
Gentamicin	Aminoglycosides	19 mm	S
Tobramycin	Aminoglycosides	19 mm	S
Chloramphenicol	Phenicols	29 mm	S
Ofloxacin	Fluoroquinolones	22 mm	S
Polymyxin B	Lipopeptides	12 mm	S
Compound sulfamethoxazole	Folate pathway inhibitors	25 mm	S

The diameters of the antimicrobial susceptibility disks were 7 mm. R, resistant; I, intermediate sensitive; S, sensitive.

Table S2. Predicted promoters in *Aeromonas* phage Ahy-yong1 genome.

Start	End	Score	Promoter Sequence
810	855	1.00	TTATTTTGCAAAGGGTATTGCAATCGGATTATGACTATGGCCTAATAGCC
821	866	0.92	AGGGTATTGCAATCGGATTATGACTATGGCCTAATAGCCCCATCGACACA
956	1001	0.96	CCAGAGTTGACACGAAAGGGGCAACGGGTTAGAGTACACAGCATGACAGG
1134	1179	0.91	GCTGAGTTGACACGGTACACCAAGTCAGCTAAGCTGGAAGCCAAGGTGCA
1407	1452	0.95	AAGCGGTTGACAGACTCGATAATCCCTGATGTAATGGGCACCACGACGGA
4488	4533	0.92	GGAGTGGAAACCATGAAACTCGTTATCCGTGAAATGTTCGTTATGGGCAA
11421	11466	0.94	TGATTGTTGACTATGAGACCCAGAACCACGAGTATCTGGGTCATGTAGCC
11513	11558	0.97	GAGTTGGTTGACTTTGGCAATGGCGCCACTGTCATCCATCCACGCTGGTT
13939	13984	0.94	ACCTTTGAAATTGTGGTGTTCACCGCAGCTATCGTAATCCTGTCCGATAA
15293	15338	0.93	GATGGTGTCGAGTGGGGCGACGACTGCGTTATCTTCTCCGAGGATAAGGA
15589	15634	0.92	CTTTATTGGGACCTCGACTGAGCTCCCGGTAGCCTACCACGTCCTGAGCG
17918	17963	0.94	ATCCTGATTGACTGCCCCAAGTACCATCTGATTATGCGGGCGCAGGACGA
21337	21382	0.99	GTGTGTTGGATAGTCCAAACTTCTTTAGATAACCTAGTTTATATAATTAG
21369	21414	0.94	CCTAGTTTATATAATTAGTTAATACCTAGTAATATACCTAGTTATTATCC
21722	21767	0.94	CAGAGTGTTGAGTGTGTCCAGCCGGCCGGTAAGTTATTCCGCATCGGCGA
29664	29709	0.92	GGGTTGATAAGTCGTTCGAGAGAGCAGAGTCTGATGCGCGTTTCATGAAC
30886	30931	0.93	GGTCGTGTTGCTACCTCCGCCGAGCAGGCTACCATCCGGCGTAGCTTGGA
36183	36228	0.93	ACACATTCGACATCAACTTCGCCGGTGGCTACATTGACCGCACGCATGTA
36398	36443	0.97	GCCAACTTGGACAGGAACGCGAAGCAGGCTGTATTCGTAGCCGCTGAAAT
37386	37431	0.93	ACGGGTTTGAGATTGACGATGGCTCGCAGGATGTTGTTGTAGACTCATGC
37396	37441	0.93	GATTGACGATGGCTCGCAGGATGTTGTTGTAGACTCATGCTATGTGTACG
37418	37463	0.95	GTTGTTGTAGACTCATGCTATGTGTACGGTAACAACAGCGGAACCAAGGC

The underlined letters are TATA boxes and Sextama boxes of predicted promoters.

Table S3. ORF analysis of the *Aeromonas* phage Ahy-yong1 genome.

ORF	Size (aa)	Prediction function	Top BLAST hit[a]	Identity[b] (aa)	E-value[c]
1	55	hypothetical protein	No hit		
2	92	hypothetical protein	ref|YP_009793000.1|hypothetical protein HOS18_gp27 [*Aeromonas* phage CF7]	87.50% (77/88)	1e-48
3	199	hypothetical protein	ref|YP_009793001.1|hypothetical protein HOS18_gp28 [*Aeromonas* phage CF7]	96.98% (193/199)	3e-135
4	51	hypothetical protein	No hit		
5	88	hypothetical protein	ref|YP_009793002.1|hypothetical protein HOS18_gp29 [*Aeromonas* phage CF7]	73.03% (65/89)	5e-37
6	354	hypothetical protein	ref|YP_009793003.1|hypothetical protein HOS18_gp30 [*Aeromonas* phage CF7]	83.90% (297/354)	0.0
7	113	hypothetical protein	ref|YP_009793004.1|hypothetical protein HOS18_gp31 [*Aeromonas* phage CF7]	100.00% (113/113)	8e-74
8	66	hypothetical protein	ref|YP_009793005.1|hypothetical protein HOS18_gp32 [*Aeromonas* phage CF7]	92.42% (61/66)	4e-33
9	152	hypothetical protein	ref|YP_009793007.1|hypothetical protein HOS18_gp34 [*Aeromonas* phage CF7]	40.00% (40/100)	4e-04
10	103	hypothetical protein	ref|YP_009793008.1|hypothetical protein HOS18_gp35 [*Aeromonas* phage CF7]	72.73% (72/99)	4e-44
11	76	hypothetical protein	ref|YP_009793009.1|hypothetical protein HOS18_gp36 [*Aeromonas* phage CF7]	82.89% (63/76)	5e-37
12	142	hypothetical protein	ref|QDH46261.1|hypothetical protein LAh1_23 [*Aeromonas* phage LAh1]	67.83% (97/143)	1e-65
13	78	hypothetical protein	ref|YP_009793011.1|hypothetical protein HOS18_gp38 [*Aeromonas* phage CF7]	56.52% (39/69)	6e-17
14	158	hypothetical protein	ref|YP_009793012.1|hypothetical protein HOS18_gp39 [*Aeromonas* phage CF7]	56.52% (91/161)	4e-48
15	122	hypothetical protein	ref|QDH46267.1|hypothetical protein LAh1_25 [*Aeromonas* phage LAh1]	73.77% (90/122)	3e-52
16	75	hypothetical protein	ref|QDH46274.1|hypothetical protein LAh1_26 [*Aeromonas* phage LAh1]	59.46% (44/74)	2e-15

17	227	DNA primase	ref|YP_009793015.1|putative DNA primase [*Aeromonas* phage CF7]	88.55% (201/227)	4e-149
18	416	DNA helicase	ref|QDH46241.1|putative DNA helicase [*Aeromonas* phage LAh1]	95.43% (397/416)	0.0
19	69	hypothetical protein	ref|YP_009793017.1|hypothetical protein HOS18_gp44 [*Aeromonas* phage CF7]	85.51% (59/69)	9e-36
20	65	hypothetical protein	ref|QDH46266.1|hypothetical protein LAh1_31 [*Aeromonas* phage LAh1]	54.55% (36/66)	2e-15
21	327	ATP-dependent DNA ligase	ref|QDH46243.1|putative ATP-dependent DNA ligase [*Aeromonas* phage LAh1]	93.88% (307/327)	0.0
22	216	nucleotidyltransferase	ref|QDH46294.1|putative nucleotidyl transferase [*Aeromonas* phage LAh2]	62.50% (120/192)	1e-80
23	828	DNA polymerase	ref|QDH46235.1|putative DNA polymerase [*Aeromonas* phage LAh1]	93.24% (772/828)	0.0
24	32	hypothetical protein	No hit		
25	291	hypothetical protein	ref|YP_009793043.1|hypothetical protein HOS19_gp21 [*Aeromonas* phage Ahp1]	89.08% (261/293)	1e-178
26	299	5'-3' exonuclease	ref|QDH46245.1|putative DNA exonuclease [*Aeromonas* phage LAh1]	96.66% (289/299)	0.0
27	141	hypothetical protein	ref|QDH46260.1|putative DNA exonuclease [*Aeromonas* phage LAh1]	84.40% (119/141)	1e-81
28	138	recombination endonuclease VII	ref|QDH46263.1|putative DNA endonuclease [*Aeromonas* phage LAh1]	94.20% (130/138)	3e-89
29	324	hypothetical protein	ref|YP_009792978.1|hypothetical protein HOS18_gp05 [*Aeromonas* phage CF7]	91.67% (297/324)	0.0
30	183	phosphatase	ref|QDH46256.1|putative kinase phosphatase [*Aeromonas* phage LAh1]	80.87% (148/183)	3e-105
31	214	deoxynucleoside monophosphate kinase	ref|YP_009793049.1|putative ATP-binding protein [*Aeromonas* phage Ahp1]	85.05% (182/214)	3e-132
32	815	DNA-dependent RNA polymerase	ref|QDH46236.1|putative DNA-dependent RNA polymerase [*Aeromonas* phage LAh1]	89.45% (729/815)	0.0
33	60	hypothetical protein	ref|QDH46273.1|hypothetical protein LAh1_43 [*Aeromonas* phage LAh1]	91.23% (52/57)	5e-29
34	147	hypothetical protein	ref|QDH46259.1|hypothetical protein LAh1_44 [*Aeromonas* phage LAh1]	53.74% (79/147)	2e-48
35	130	hypothetical protein	ref|YP_009793053.1|hypothetical protein HOS19_gp31 [*Aeromonas* phage Ahp1]	90.77% (118/130)	2e-79

36	500	portal protein	ref|QDH46240.1|putative portal protein [*Aeromonas* phage LAh1]	94.60% (473/500)	0.0
37	298	capsid assembly protein	ref|YP_009792986.1|putative scaffolding protein [*Aeromonas* phage CF7]	68.12% (203/298)	2e-105
38	338	major capsid protein	ref|QDH46242.1|putative major capsid protein [*Aeromonas* phage LAh1]	94.67% (320/338)	0.0
39	191	tail tubular protein A	ref|QDH46254.1|putative tail tubular protein A [*Aeromonas* phage LAh1]	87.43% (167/191)	2e-114
40	850	tail tubular protein	ref|QDH46234.1|putative tail tubular protein B [*Aeromonas* phage LAh1]	88.13% (750/851)	0.0
41	262	hypothetical protein	ref|QDH46248.1|hypothetical protein LAh1_6 [*Aeromonas* phage LAh1]	87.34% (207/237)	8e-140
42	739	hypothetical protein	ref|QDH46237.1|hypothetical protein LAh1_7 [*Aeromonas* phage LAh1]	75.14% (556/740)	0.0
43	1253	transglycosylase	ref|QDH46233.1|putative transglycosylase [*Aeromonas* phage LAh1]	88.59% (1110/1253)	0.0
44	938	tail spike protein	ref|YP_009793062.1|putative tail fiber protein [*Aeromonas* phage Ahp1]	68.08% (145/213)	2e-71
45	60	holin	ref|YP_009792994.1|putative holin protein [*Aeromonas* phage CF7]	93.33% (56/60)	7e-31
46	121	terminase small subunit	ref|YP_009792995.1|putative DNA maturase A [*Aeromonas* phage CF7]	92.56% (112/121)	7e-75
47	641	terminase large subunit	ref|YP_009793065.1|putative DNA maturase B [*Aeromonas* phage Ahp1]	96.41% (618/641)	0.0
48	129	hypothetical protein	ref|QDH46262.1|hypothetical protein LAh1_13 [*Aeromonas* phage LAh1]	46.04% (64/139)	7e-19
49	180	lysozyme	ref|YP_009792998.1|putative endolysin [*Aeromonas* phage CF7]	97.22% (175/180)	4e-128
50	111	hypothetical protein	ref|QDH46269.1|hypothetical protein LAh1_15 [*Aeromonas* phage LAh1]	71.17% (79/111)	3e-38
51	37	hypothetical protein	ref|QDH46272.1|hypothetical protein LAh1_16 [*Aeromonas* phage LAh1]	72.97% (27/37)	7e-11
52	55	hypothetical protein	No hit		

[a] the most closely related protein and its organism. ''No hits'' indicates no significant hits detected for a particular amino acid sequence

[b] percent identity for top hits in BLASTp searches. Numbers in parentheses provide length of each alignment

[c] the probability of obtaining a match by chance as determined by BLASTp analysis

Table S4.Taxonomic status or morphological characteristic of the phages against *Aeromonas hydrophila*.

Phages	Taxonomic status or morphological characteristic
**CF7**, **Ahp1**, **LAh5**, **LAh4**, **LAh3**, **LAh2**, **LAh1**, HJG, PZL-Ah152, ZPAH7B, PZL-Ah8, PZL-Ah1, MJG, ZPAH7, 25AhydR2PP	*Autographiviridae* (family)
PVN05, PVN04, PVN03, pAh6-C, PVN02, pAh6.2TG	*Chaseviridae* (family)
AhSzw-1, AhSzq-1, Akh-2	*Demerecviridae* (family)
Aeh1, CC2, 60AhydR15PP, 50AhydR13PP, Ah1	*Straboviridae* (family)
BUCT551, LAh_7, vB_AhyS-A18P4	*Casjensviridae* (family)
LAh10, 4_L372XY, 4_L372D, 2_L372X, 2-L372D	*Plateaulakevirus* (genus)
LAh_9, LAh_8, LAh_6, 4_4572	*Lahexavirus* (genus)
D6, D3, D9, ZPAH1, AhyVDH1, CF8, PS2, PS1, AHP-1, AP1, 14AhydR10PP, 85AhydR10PP, 62AhydR11PP, 13AhydR10PP, Ahp2, AhMtk13a, PM2, pAh1-C, pAh-1, Aeh2, Φ2, Φ5	*Myoviridae*-like (unclassified)
BUCT552, 4_L372X, AhMtk13b, PM3	*Siphoviridae*-like (unclassified)
CT45P, TG25P	Unknown

The top 7 BLASTn hits having the highest coverage with *Aeromonas* phage Ahy-yong1 were showed in bold.

Table S5. The ANI value, isDDH value and intergenomic similarities between *Aeromonas* phage Ahy-yong1 and the 7 most related *Aeromonas* phages.

Aeromonas phages	ANI value (%)	isDDH value (%)	intergenomic similarities (%)
Ahy-yong1 and CF7	82.23	26.40	75.20
Ahy-yong1 and Aph1	82.46	25.30	72.40
Ahy-yong1 and LAh1	82.26	27.40	73.20
Ahy-yong1 and LAh2	82.24	27.30	73.20
Ahy-yong1 and LAh3	82.25	27.30	73.20
Ahy-yong1 and LAh4	82.25	27.30	73.20
Ahy-yong1 and LAh5	82.19	27.40	73.20

Table S6. Comparison of the characteristics of *Aeromonas* phage Ahy-yong1 and the closet relatives.

Phages	Genome size (bp)	G+C (%)	ORFs	Head diameter (nm)	Tail length (nm)	Host range	Adsorption rate in 2 min	Burst size (PFU/ cell)	Biofilm removal capacity
Ahy-yong1	43374	59.4	52	66	26	*A. hydrophila* A18	99.75% ± 0.03%	637 at MOI of 0.1; 3952 at MOI of 0.01	Yes
Aph1	42167	58.8	46	62	12.5	*A. hydrophila* ATCC 7966, H6, H10, H23, H30 and H32	96%	112 at MOI of 0.0001	Unknown
LAh1	42002	59.3	45	82.3	8±1	*A. hydrophila* AHB0147	Unknown	Unknown	Yes
LAh2	42008	59.3	45	82.38	8±1	*A. hydrophila* AHB0147	Unknown	Unknown	Unknown
LAh3	42002	59.3	50	82.4	8±1	*A. hydrophila* AHB0147	Unknown	Unknown	Unknown
LAh4	42002	59.3 %	52	82.4	8±1	*A. hydrophila* AHB0147	Unknown	Unknown	Unknown
LAh5	41985	59.3	53	82.4	8±1	*A. hydrophila* AHB0147	Unknown	Unknown	Unknown
CF7	42439	59.0	49	Unknown	Unknown	Unknown	Unknown	Unknown	Unknown
